# Central and peripheral plasticity after chemical unilateral labyrinthectomy: Glial responses, calyces dynamics, and behavioral consequences

**DOI:** 10.1371/journal.pone.0355839

**Published:** 2026-09-01

**Authors:** Jessica Trico, Isabelle Watabe, Vinay Parameshwarappa, Agnes Lapotre, Louis Godaert, Alain Tonetto, Christian Chabbert, Andreas Zwergal, Brahim Tighilet

**Affiliations:** 1 Aix Marseille Univ, CNRS, CRPN (Centre de Recherche en Psychologie et Neurosciences UMR 7077), Marseille, France; 2 Aix Marseille Univ, CNRS, Centrale Med, FSCM (UAR1739), Plateforme de Recherche Analytique Technologique et Imagerie (PRATIM), Marseille, France; 3 Department of Neurology, LMU University Hospital, LMU Medizin, LMU Munich, Munich, Germany; 4 German Center for Vertigo and Balance Disorders (DSGZ), LMU University Hospital, LMU Medizin, LMU Munich, Munich, Germany; LSU Health Shreveport, UNITED STATES OF AMERICA

## Abstract

Peripheral vestibular disorders are highly prevalent, but the underlying neurobiological mechanisms remain poorly understood, particularly regarding bilateral peripheral plasticity following unilateral injury. Using a rat model of chemical unilateral labyrinthectomy (cUL) (SHAM, n = 14; cUL, n = 20), we combined behavioral analyses, auditory assessments, immunohistochemistry, and histological quantification to characterize central and peripheral adaptations from 1 to 30 days after vestibular deafferentation. cUL induced significant postural deficits from day 1, while hyperactivity and anxiety-like behaviors emerged from day 9 and persisted over time. In the deafferented medial vestibular nucleus, robust glial reactivity developed rapidly (day 3) whereas only astrocytes remained significantly elevated throughout the observation period. Histological analyses revealed a marked loss of type I hair cell calyces in the ipsilateral utricle to ototoxic exposure at both acute (D3) and chronic (D30) time points. Unexpectedly, the contralateral utricle also exhibited a transient reduction in calyceal endings at D3 (p < 0.01), which recovered by D30, revealing previously unrecognized bilateral peripheral vestibular plasticity following unilateral vestibular injury. In contrast, cochlear alterations remained restricted to the ipsilateral side of ototoxic exposure indicating distinct adaptive responses in the auditory and vestibular organs. Together, these findings demonstrate that unilateral vestibular injury triggers coordinated central glial reactivity and contralateral peripheral vestibular plasticity, providing new insight into the cellular mechanisms underlying vestibular compensation.

## Introduction

Peripheral vestibular disorders are among the most common causes of dizziness in the general population and their prevalence continues to rise with aging [1,2]. Despite significant efforts to create animal models for these disorders [3–10], available treatment options remain limited or are non-specific [11]. This underscores the need for a deeper understanding of the cellular and molecular mechanisms involved.

Among all vestibular disorders, acute unilateral vestibulopathy is consistently reported as one of the leading causes of acute peripheral vestibular syndrome [12,13]. Complete recovery from symptoms of acute unilateral vestibulopathy depends both on successful central compensation of static vestibular tone imbalance, as well as peripheral plasticity and repair to reinstall dynamic vestibular function [14]. In clinical cohorts, about 15–25% of patients will show an incomplete peripheral repair one year after onset of acute unilateral vestibulopathy [15,16] and about 10–30% a persistently incomplete central vestibular compensation [17]. The underlying factors for the individual variability of outcomes are not fully understood. It is hypothesized that older age, lack of exercise, other sensory comorbidities (e.g., poor vision, polyneuropathy) and intake of centrally acting drugs (e.g., sedatives, neuroleptics) could be risk factors for incomplete compensation and recovery. However, it is not clear, how these factors biologically would impair adaptative peripheral and central neuroplasticity after acute unilateral vestibulopathy.

Animal models of unilateral peripheral vestibular loss, such as the chemical unilateral labyrinthectomy (cUL) model induced by transtympanic injection of arsanilic acid, may help to better understand the cellular and molecular processes of central vestibular compensation and peripheral vestibular plasticity and their interference with modulatory factors. The cUL model in rats has provided insights into locomotor deficits and recovery dynamics [18–22], while postural disturbances are still poorly documented. The central plasticity following cUL has revealed glial reactivity in the vestibular nerve and vestibular nuclei (VN), depicted in vivo by micro-positron emission tomography [10], yet the contribution of astrocytes, microglia, and oligodendrocytes has not been examined. While glial plasticity and neurogliogenesis have an important role after vestibular nerve section [23,24], it is still unknown, whether similar responses occur following cUL, which is a less severe, progressive and preganglionic vestibulopathy model. Additionally, while contralesional VN are now known to undergo robust post-lesional plasticity and can no longer be considered a true control region [25,26], the contralateral ear is still widely used as a peripheral reference after unilateral vestibular injury [9,27–29]. Thus, potential contralesional peripheral plasticity remains largely unexplored.

In this study, we aim to characterize the adaptive neurobiological mechanisms triggered by an ototoxic exposure leading to cUL, combining analyses of postural and anxiety-like behaviors with cellular investigations in both the central and peripheral vestibular systems. We will evaluate structural damage in the utricle and cochlea of both ears at defined postoperative delays, with a particular focus on type I hair cell calyces, which are more susceptible to ototoxic damage in patients [30–32]. Simultaneously, we will investigate glial response and cell proliferation in the deafferented medial vestibular nucleus (MVN) to better understand the central response to vestibular loss in this ototoxic model.

## Materials and methods

### Animals and ethical statement

34 female Long Evans rats (9−10 weeks, 200–300 g) originating from Charles River (St Germain sur l’Arbresle, France) were used for the experiments. All experiments were performed in accordance with the European Union 2010/63/EU Directive and under veterinary and National Ethical Committee supervision (French Agriculture Ministry Project Authorization: APAFIS #40187−2022120518126271). The present study was approved by Neurosciences Ethic Committee N°71 of the French National Committee for animal experimentation. All personnel participating in this study were trained in animal welfare and care and possessed the mandatory French certification for animal experimentation. All surgeries were performed under isoflurane or ketamine and medetomidine anesthesia depending on the surgery (see either chemical unilateral labyrinthectomy section and auditory brainstem response test section). Humane endpoints—based on grooming, natural behavior, hydration status, clinical signs such as breathing/ventilation, response to handling, and anorexia (> 20% loss of pre-operative body weight)—triggered immediate euthanasia. No animal in this study reached these endpoints, and all animals were perfused at the end of the experiment for immunohistochemical analysis. Every attempt was made to minimize both the number and suffering of the animals used in the experiment. The animals were housed at the Centre de Recherche en Psychologie et Neurosiences (Aix Marseille University) animal facility, with 12 h – 12 h diurnal light variations and free access to water and food.

### Study design

For vestibular assessment, 28 rats were assigned to two groups (SHAM, n = 12 and cUL, n = 16) for behavioral and immunohistochemical assessment and were manipulated for 14 days prior to the ototoxic exposure. Among them, one animal was excluded from the D30 cUL group (see Criteria for exclusion section). Thus, 23 female rats (SHAM, n = 12 and cUL, n = 11) were used for behavioral assessment and killed 30 days after cUL for immunohistochemical analysis (see below). To monitor vestibular dysfunction, a qualitative assessment was conducted at a preoperative session and daily for the first 10 days post-cUL, and subsequently on D15, D21, and D30, to define the acute, post-acute, and compensated phases of the vestibular syndrome. Based on the time course of vestibular deficits observed in this qualitative assessment, time points for the postural assessment (Dynamic Weight Bearing 2) [33] and light/dark box test (LDBT) were selected to capture key stages of behavioral recovery. These tests were therefore performed at preop, D1, D4, D7, D9, D15, D21, and D30 postop.

**Fig 1:**
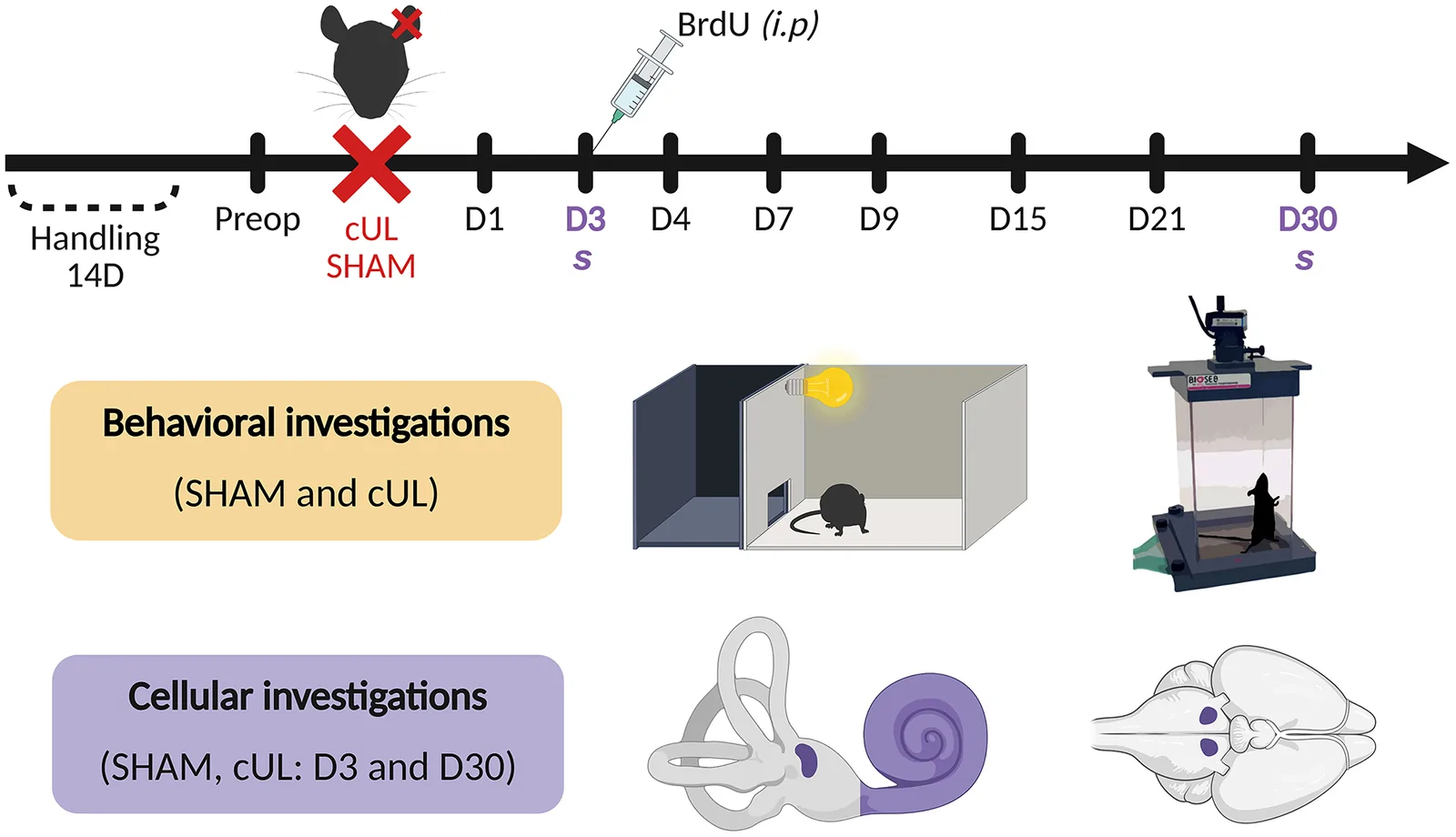
Study design used to assess the behavioral and cellular effects of chemical unilateral labyrinthectomy (cUL). In total n = 12 animals received a SHAM and n = 16 rats a cUL surgery. The peripheral vestibular system (Scarpa’s ganglion and utricle), cochlea and central vestibular nuclei (medical vestibular nuclei, MVN) were studied at D3 (n = 4, D3 cUL group) and D30 (n = 5, SHAM group and n = 4, cUL group) time points. cUL group on D3 received an injection of BrdU (bromodeoxyuridine, 200 mg/kg, i.p) and was killed either 3 h after the injection (n = 4) or 30 days after surgery (n = 4) to study acute cell proliferation and long-term plasticity. SHAM group on D3 received the same BrdU injection but was killed only 30 days after surgery (n = 5). Quantitative assessment of vestibular syndrome and anxiety-like behavior were performed one day before the surgery and 1, 4, 7, 9, 15, 21, 30 days after the surgery in SHAM (n = 12) and cUL (n = 11) group. s: sacrifice.

For immunohistochemical analysis, the peripheral vestibular system (Scarpa’s ganglion and utricle), cochlea and central vestibular nuclei were collected at D3 (D3 cUL, n = 4) and D30 (SHAM, n = 5 and D30 cUL, n = 4). All cUL animals on D3 received an injection of BrdU (bromodeoxyuridine, 200 mg/kg, i.p) and were killed either 3 h after the injection (n = 4) or 30 days after surgery (n = 4) to study acute cell proliferation and long-term plasticity. All SHAM animals on D3 received the same BrdU injection but was killed only 30 days after surgery (n = 5) (Fig 1).

For auditory assessment, 6 additional rats (SHAM, n = 2 and cUL, n = 4) were used to analyze auditory bainstem responses (ABR) at preop, D3 and D30 postop.

### Chemical unilateral labyrinthectomy

cUL group (n = 20) was subjected to a left-side chemical unilateral labyrinthectomy, following the surgical procedure previously reported in the literature [10,19,34,35]. Thirty minutes after a buprenorphine (Buprecare®, 0.03 mg/kg, s.c.) injection, the rats were anesthetized with isoflurane (induction 5% for 4 minutes, mask 2.5%). After local anesthesia with 1% lidocaine (0.1 mL, s.c.), a left paramedian incision was made to expose the lamboidal ridge and the external ear canal. The external ear canal was opened just anterior to the exit point of the facial nerve. With homemade forceps, the tympanic membrane was perforated caudally to the hammer shaft, and 150 µL of a 20% bupivacaine solution was instilled into the tympanic cavity with a 26-gauge needle. During about 3 min, the bupivacaine solution was aspirated and instilled slowly again. After the local anesthetic was instilled, the same procedure was performed to instill 150 µL of a 10% solution of p-arsanilic acid during about 4 min. After the last thorough aspiration, the wound was closed by 3 stitches (vicryl 3−0). Before awakening, a solution of Ringer Lactate (Virbac, 10 mL/kg, s.c.) was injected to alleviate the dehydration resulting from the rats’ inability to drink normally due to the ototoxic exposure. Post-operatively, animals were monitored daily for 1 week, corresponding to the critical phase of the vestibular syndrome, and high-fat food was provided in the cage to compensate for food intake and support recovery. Body weight and general condition were assessed daily using a standardized clinical scoring grid throughout this period. The success of the procedure was attested upon awakening by the onset of characteristic vestibular symptoms. In the SHAM surgery group (n = 12), 150 µL of 0.9% saline was instilled into the tympanic cavity during 7 min.

### Criteria for exclusion

Animals were excluded from the study if no typical vestibular syndrome was observed at awakening or if the following symptoms were observed:

- loss of body weight equal to more than 20% of the pre-treatment,
- ulcer of the cornea, which could occur due to an inadvertent lesion of the facial nerve,
- bleeding from the tympanic cavity, which could prevent the diffusion of bupivacaine or p-arsanilic acid into the inner ear,
- abnormality in behavioral qualitative scoring, e.g., convulsions, paresis, or hemiataxia.

Following these criteria, one animal has been excluded from the D30 cUL group because no typical vestibular syndrome was observed at awakening, resulting in n = 11 animals in the D30 cUL group included in the study for behavioral analysis.

### Auditory brainstem responses (ABR)

The hearing thresholds before, 3 days, and 30 days after surgery were assessed using auditory brainstem responses (ABR). The ABR recordings were collected using needle electrodes positioned under the skin, at the skull vertex (active electrode), behind left or right mastoid (reference electrode), and in the neck muscle (ground electrode). Signals were amplified 10^4^ times, filtered between 300 and 3000 Hz (Grass ICP 511 amplifiers), digitally converted, and averaged with a Micro1401 Plus system (Cambridge Electronic Devices, UK). Auditory stimuli were tone pips (with 2ms linear rise/fall time with no plateau) at octave frequencies from 2 to 32 kHz at a repetition rate of 10 s^-1^ presented via an in-ear miniature earphone placed in one ear at a time. Tone pip level varied from 90 dB SPL to 0 dB SPL using 10-dB steps. For each ear, stimulus repetitions incremented from 200 at 90 dB SPL to 1800 at 0 dB SPL. ABR were analyzed offline using a custom-written MATLAB program. The five ABR wave maxima correspond to the different brainstem nuclei along the auditory pathway starting from the auditory nerve (wave I) to the inferior colliculus (wave V). The threshold was estimated as the lowest intensity of stimulation that yielded a repetitive waveform. Threshold shifts were evaluated by subtracting the ABR threshold obtained before surgery from the threshold obtained 3 and 30 days after surgery.

### Behavioral assessment

#### Qualitative assessment of vestibular syndrome.

Behavioral symptoms of vestibular imbalance following cUL were scored for 9 components (adapted from [7,36]).

- Barrel rolling, corresponds to a rotation of the rat along its body axis, was scored from 0 (absence of barrel rolling) to 2 points (presence of barrel rolling)
- Circling, relates to circulars movements of the rats in the horizontal plane, was scored from 0 (absence of circling), 1 (1/2 circling or 1 circling), 2 (at least two followed circling) to 3 points (multiple successive circling)
- Bobbing, describes as rapid head tilts to the rear and is assimilated to cephalic nystagmus, was scored from 0 (absence of bobbing) to 1 point (presence of bobbing)
- Headtilt, characterizes an inclination of the head relative to the body axis, was scored from 0 (absence of headtilt) to 1 point (presence of headtilt)
- Support surface area, assimilated to the surface area delimited by the 4 rats’ paws, was scored from 0 (small support surface area), 0.5 (mild support surface area), 1 (large support surface area) to 2 points (enlarged support surface area with abdomen on the floor)
- Rearing, describes the rats’ ability to stand in two rear legs, was scored from 0 (presence of rearing) to 1 point (absence of rearing)
- Displacement, was scored from 0 (displacement of the rat with non-visible deficit), 1 (slight instability while walking), 2 (tortuous displacement) to 3 points (tortuous displacement and fall)
- Tail hanging behavior was assessed by picking up the animal at the base of the tail and scoring body rotation from 0 (absence of rotation), 0.5 (twist), 1 (rotation during one trial) to 2 points (rotation during the two trials)
- Landing reflex was assessed after picking up the animal from the ground and scoring it landing from 0 (presence of landing reflex), 0.5 (altered landing reflex), 1 (absence of landing reflex during one trial) to 2 points (absence of landing reflex during the two trials). When lifted by the tail, SHAM rats exhibit a landing reflex, consisting of forelimb extension, that allows them to land successfully (i.e., they land on all four legs). Rats with impaired vestibular function do not exhibit a forelimb extension, they spin or bend ventrally, sometimes “crawling” up toward their tails, causing them to miss their landings.

This qualitative assessment was conducted the day before the ototoxic exposure (preop), then daily for the first 10 days post-cUL, and subsequently on D15, D21, and D30 post-cUL.

#### Quantitative evaluation of vestibular syndrome.

The dynamic weight-bearing (DWB2) device (Bioseb, Vitrolles, France) was used to assess the postural deficits (in static and dynamic positions) of both cUL and SHAM groups as previously described in the literature [33,37]. Briefly, it consists of a Plexiglas chamber (25 x 25 cm) with a floor covered by force sensors connected to a computer along with a camera. The latest DWB2 software version available at the time (BIO-ADWB2 2.5.1) and home-made Scilab script allowed us to extract a wide variety of parameters:

- The support surface area, which is the area delimited by the 4 paws of the animal during a session
- The ellipse (or body sway) corresponds to the average area of the 90% confidence ellipse calculated from the barycenter positions. It is a classical index of postural stability in posturology
- The time spent in front left paw (ipsilateral to ototoxic exposure)
- The weight distribution on the left paws (static and dynamic) estimates lateral axis imbalance. In SHAM animals, this percentage is equally distributed between right and left
- The number of circling (left and right), where complete and fast lap made by the animal is counted
- The time spent rearing is defined as the total duration during which the animal remains on its two hind paws

This quantitative assessment was conducted the day before the lesion (preop), then on D1, D4, D7, D9, D15, D21, and D30 post-cUL or SHAM surgery.

#### Evaluation of anxiety like behavior during vestibular syndrome.

The light/dark box test is commonly used to explore anxiety-like behavior. This test relies on a conflict between exploratory behavior and the natural tendency to avoid exposed, novel and brightly lit environment. As found in the literature [38,39], the open-topped test box was home-made of PVC and divided into two compartments illuminated by a cold light and connected by a small opening (7.5 × 7.5 cm). The intensity of the light was 350 lux in the white compartment (54 x 36 x 37 cm) and 70 lux in the black compartment (27 x 36 x 37 cm). Each rat was placed at the center of the black compartment facing the opening to avoid freezing in the light compartment the first days after the vestibular lesion. Two parameters were observed for 5 min: the number of entries into the white compartment and the time spent in the white compartment. This assessment was conducted the day before the ototoxic exposure (preop), then on D1, D4, D9, D15, D21, and D30 post-cUL or SHAM surgery.

### Cellular investigations

#### Tissue preparation.

The rats were injected with buprenorphine (Buprecare®, 0.03 mg/kg, s.c.) and deeply anesthetized with a mixture of ketamine 1000 (100 mg/kg, i.p) and medetomidine (0,5 mg/kg, i.p) prior to intracardiac perfusion. The intracardiac injection of 400 mL of isotonic saline (0.9% NaCl) was followed by 400 mL of 4% paraformaldehyde in 0.1 M phosphate buffer (PB), pH 7.4. At the end of the perfusion, two different samples were collected: the brain and the two temporal bones.

The brain was postfixed overnight at 4°C in the same fixative solution as used during the perfusion, then cryoprotected in sucrose solution (10, 20, 30% in 0.1 M PB) for 72 h at 4°C. It was subsequently frozen in isopentane and cut into 40µm serial frontal section with a cryostat (Leica, Wetzlar, Germany) for immunochemistry. Based on the rat brain stereotactic atlas [40], sections encompassing the full rostrocaudal extent of the VN (−9.84 mm to −13.08 mm from Bregma) were collected. Sequential sections were systematically distributed into a 12-well plate containing cryoprotectant solution, so that each well contained a series of 10–11 sections evenly spaced and covering the full extent of the VN.

The temporal bones were postfixed at room temperature for 30 min in the same fixative solution used during perfusion, then rinsed (0.1 M PB). The Scarpa’s ganglions were isolated, and the inner ears were microdissected at room temperature to collect the cochleas and utricles. Scarpa’s ganglions were embedded in OCT compound and cryosectioned onto slides (10µm) and stored at −80°C until staining. Cochleas and utricles were preserved at −20°C in a cryoprotectant solution until whole-mount immunostaining.

#### Immunohistochemistry.

For brain slices, immunochemical labeling was performed according to previously validated protocols [36,41,42]. Cell proliferation was analyzed in D3 cUL after injection of BrdU on D3 post-cUL, and the animals were killed 3 h later. Survival and differentiation of the newly generated cells were analyzed in groups SHAM and D30 cUL that were injected with BrdU on D3 post-surgery and killed on D30. Floating brain sections were washed (3 x 5 min) with PBS in multi-well plates. Blocking was done by incubation (2 h, RT) in 10% SVF and DMEM. Then, heat-induced epitope retrieval was performed with 2M HCl in PBS 0.5% Triton X-100 (30 min, 37°C), followed by neutralization with sodium tetraborate (pH = 8.5, 3x 5 min, RT). The pH of the free-floating sections was then verified using DMEM with pH indicator, then rinsed with PBS (3 x 5 min, RT) and incubated overnight at 4°C with primary antibody mouse anti-BrdU (1:100, Dako, M0744) or mouse anti-BrdU (1:200, Proteintech, 66241–1-IG). Alexa fluor 594 nm donkey anti-mouse secondary antibody was then used (1:500, Invitrogen, A21203) for 2 h at room temperature. Finally, brain sections were mounted onto SuperFrost/Plus glass slides (Epredia, J1800AMNZ) and air-dried before being mounted with Roti®Mount FluorCare antifade reagent with the nuclear marker DAPI (Carl Roth, Karlsruhe). For other immunostaining, floating brain sections were washed (3 x 5 min) with PBS in multi-well plates and blocking was done by incubation (1 h, RT) in 5% BSA and 0.3% Triton X-100. Floating brain sections were incubated overnight at 4°C with the following primary antibodies: rabbit anti-IBA1 (1:2000, Fujifilcdi, 019–19741), rabbit anti-Olig2 (1:500, Millipore, AB9610), rabbit anti-GFAP (1:200, Dako, Z0334), mouse anti-GFAP (1:200, Invitrogen, MA5–12023), rabbit anti-KCC2 (1:500, Millipore, 07–432). Fluorescent secondary antibodies were used as follows: Alexa fluor 488 nm donkey anti-rabbit (1:500, Invitrogen, A21206) and Alexa fluor 594 nm donkey anti-mouse (1:500, Invitrogen, A21203) for 2 h at room temperature and brain sections were mounted as described previously.

Scarpa’s ganglion sections were stained with methylene blue (3 min) using classical protocol to visualize cell morphology.

The whole-mount immunostaining was performed as previously described for utricles and cochleas [43]. Tissues were first incubated for a 15 min period in citrate sodium buffer (pH 6, 10 mM in 0.1 M PBS) at 95°C and rinsed for three-time 10 min period in 0.1 M PBS. They were then left for 90 min in 0.1 M PBS with 4% Triton X-100 and 5% donkey serum under slow agitation. The primary antibodies were incubated in 0.1% Triton X-100 and 1% donkey serum in 0.1 M PBS for 24 h at 4°C under slow agitation: rabbit anti-Myo7a (1:200, Life technologies, PA 1936), goat anti-NKAα3 (1:250, Santa cruz, sc-16052), mouse anti-NKAα3 (1:250, Santa cruz, sc-365744). After three washes (10 min, 0.1 M PBS), the secondary antibodies were incubated in 0.1% Triton X-100 and 1% donkey serum in 0.1 M PBS overnight at 4°C: Alexa fluor 488 nm donkey anti-rabbit (1:500, Invitrogen, A21206), Alexa fluor 647 nm donkey anti-goat and (1:500, Invitrogen, A21447) and Alexa fluor 594 nm donkey anti-mouse (1:500, Invitrogen, A21203). A chromatin staining with DAPI (1:5000, Invitrogen, D1306) was performed between the two final washes. The epithelia were oriented and mounted in Mowiol medium.

#### Fluorescence utricle image acquisition.

Fluorescence images were acquired using a Zeiss (LSM 710 NLO) confocal microscope with a 63 × Zeiss Plan Apochromat oil-immersion lens (NA 1.40, DIC M27) controlled with the Zen software (ZEN 2012 Black edition, Carl Zeiss Microscopy GmbH, Germany). We acquired *z*-stacks (12–30 optical sections) through the majority of preparations that we collected in 0.772 µm steps. The step size (optical section thickness) was determined by stepping at half the distance of the theoretical *z*-axis resolution. Images were acquired to a resolution of 1024 × 1024 pixels at subsaturating laser intensities for each channel.

#### Cells count.

NKAα3 specifically labels peripheral vestibular afferents [44]. While it is expressed in both type I and type II endings (calyx and bouton afferents, respectively), type II labeling is punctate, whereas type I labeling forms a continuous membrane ring extending to the apical pole of the hair cell [44]. We therefore quantified only complete NKAα3 labeling around DAPI-positive nuclei, providing a quantification of functionally innervated type I hair cells. Calyx quantification in the utricle was performed under blinded and randomized conditions. For calyx quantification in the utricle, ROI in the extrastriolar or striola were randomly chosen and only structures displaying a calyx-like morphology were included: cylindrical structures with an apical constriction and a visible nucleus, fully enclosing the ciliated cell at the level of the nucleus. The entire z-stack was examined to avoid duplicate counts of the same cell.

For quantification of cells expressing specific markers in the vestibular nuclei, 1 in 12 serial sections starting at the beginning of the vestibular nuclei (relative to bregma, −9.84 mm) to the end of the vestibular nuclei (relative to bregma, −13.08 mm) were used, according to previously validated protocols [36,42]. Only sections of the MVN on the deafferented (left) side were evaluated. Quantification of BrdU+ , IBA1+ , GFAP+ , Olig2+ cells was counted using a confocal imaging with a Zeiss LSM 710 NLO laser scanning microscope. For each animal, about 10 coronal sections of the deafferented MVN were analyzed. For each marker, immunoreactive positive cells in the deafferented MVN were counted using an integrated microscopic counting chamber that delineated the region of interest by a square of 425.10 μm^2^. The average cell counts from the sections were used for statistical analysis.

#### Quantification of IBA1+ and GFAP+ fluorescence area, and KCC2 fluorescence intensity.

To calculate immunofluorescence area of IBA1+ and GFAP+ immunostaining we used the ImageJ analyze particle plugin after thresholding the image.

To analyze the neuronal membrane fluorescence of KCC2, a custom program written in Matlab® (Mathcorks, Incs) was used as previously described [36,45]. Briefly, non-specific immunofluorescence was estimated in a neuron-free region to define a threshold (mean fluorescence + 3 times the standard deviation). Only signal above this threshold was analyzed. A region of interest was then drawn around plasma membrane of neurons and mean membrane fluorescence was measured over pixels exceeding 20% of the maximum value, to quantify level of neuronal KCC2 membrane expression in the lateral vestibular nucleus.

### Statistical analysis

We performed all statistical analysis using GraphPad Prism software (version 10, GraphPad Software). Summary graphs are all shown as mean ± SEM. A two-way repeated measures ANOVA with two post-hoc tests were used for behavioral data. Sidak’s was used to determine statistical differences between the groups and Dunnett’s multiple comparison to compare values at each postoperative time with the preoperative value (delay effect). A two-way repeated measures ANOVA with Tukey’s post-hoc tests was used for the threshold shift analysis. Sphericity Greenhouse-Geisser correction has been applied to the data set when the normality was not verified. The statistical analyses of cellular data in the deafferented MVN were evaluated by nested one-way ANOVA followed by post hoc analysis with Tukey’s test. Kruskal Wallis’ test and Dunn’s multiple comparison post hoc test were used for calyx counting in the utricle. To determine the number of animals used for the behavioral assessment in this study, we conducted a power analysis with the G × Power software. We set the effect size at 0.25, the alpha at 0.05 and the power (1-beta) at 0.95 with a group number of 2 and 8 measurements throughout time. The power analysis gives us a total sample size of 24 and therefore, we used 24 rats in total for the behavioral assessment, but one rat was excluded (see section 5.4). p values of < 0.05 were considered statistically significant. * p < 0.05, ** p < 0.01, *** p < 0.001.

## Results

### Chemical unilateral labyrinthectomy induces long term peripheral vestibular alteration

**Fig 2:**
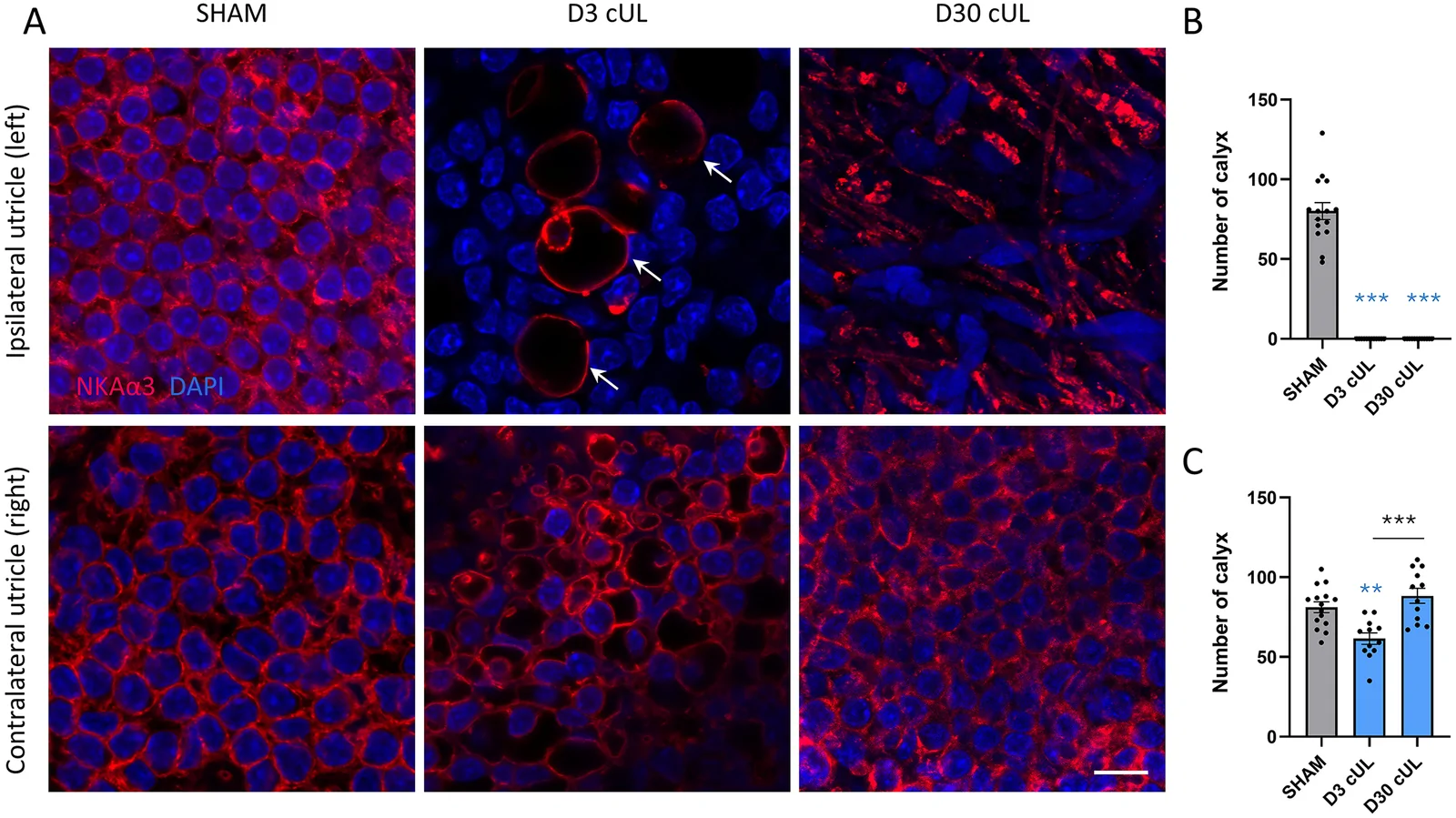
Chemical unilateral labyrinthectomy permanently destroys calyces in the utricle ipsilateral to ototoxic exposure. A. Confocal immunostaining images of NKAα3+ (red) and DAPI (blue) in the utricles ipsilateral (left) and contralateral (right) to ototoxic exposure for SHAM group (n = 5), three days (D3 cUL, n = 4) and thirty days (D30 cUL, n = 4) after cUL. Scale bar = 10 µm. Quantitative assessment of the cUL effect on the number of NKAα3 + calyces in the ipsilateral utricle (B) and in the contralateral utricle (C) relative to ototoxic exposure. The histogram represents group means ± SEM, and each point corresponds to one analyzed z-stack image, with three images analyzed per animal. A significant difference from the SHAM group is indicated by * in blue for D3 cUL and D30 cUL groups. A significant difference between the D3 cUL and D30 cUL group is indicated by * in black (** p < 0.01, *** p < 0.001; Kruskal Wallis’ and Dunn’s multiple comparison post hoc tests).

**Fig 3:**
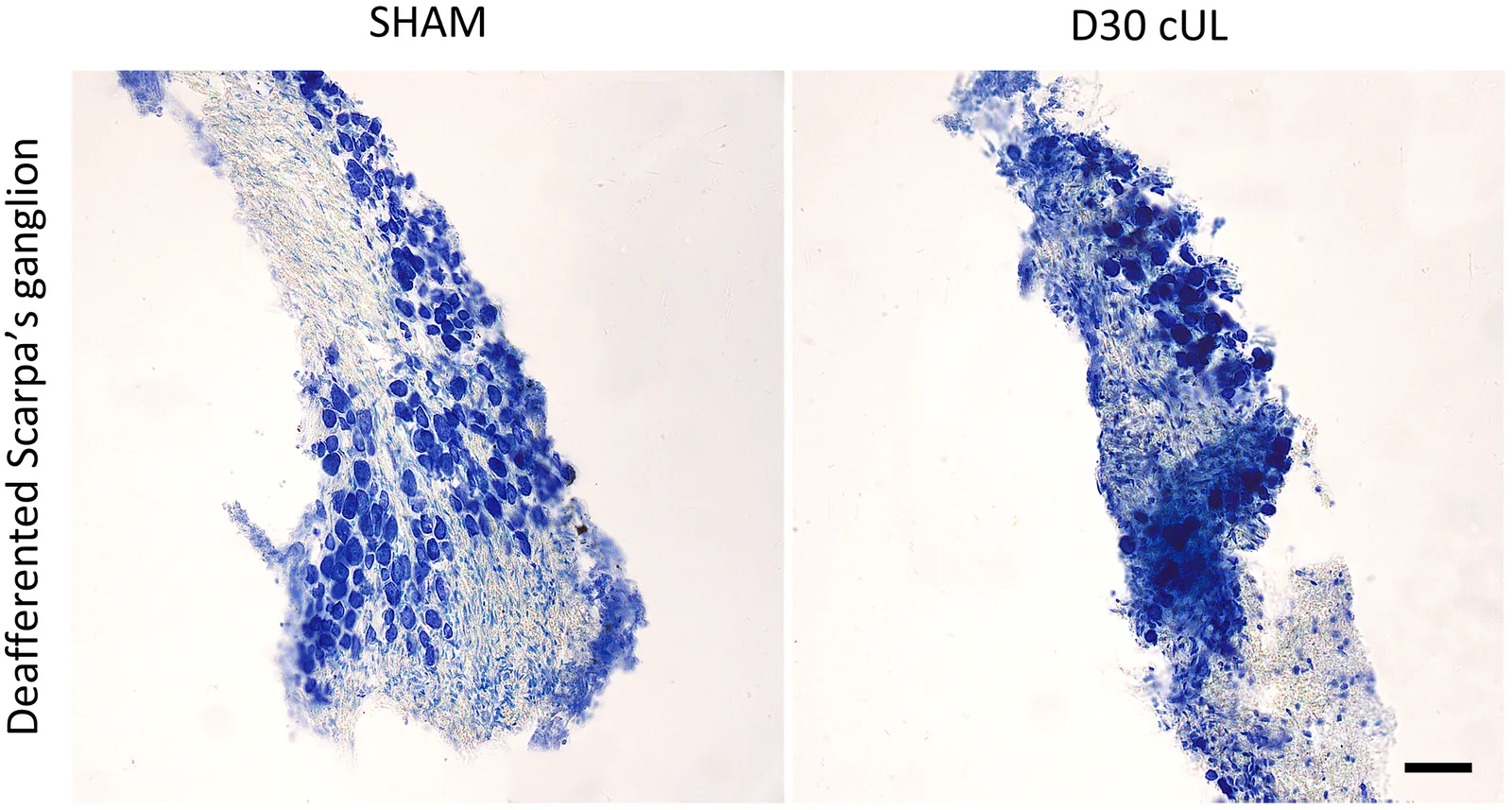
Chemical unilateral labyrinthectomy preserves Scarpa’s ganglion. Illustration of methylen blue in the left Scarpa’s ganglion of the SHAM (n = 5) and D30 cUL (n = 4) groups. Scale bar = 50 µm.

The peripheral vestibular system detects environmental stimuli notably through the utricle, an otolith organ which contains type I hair cells surrounded by calyces (Fig 2A). These calyces relay information to the vestibular nuclei via the Scarpa’s ganglion. Calyces were entirely lost on the side exposed to p-arsanilic acid (left) as early as D3 post-cUL and remained permanently absent at D30 post-cUL, compared to SHAM (*** p < 0.001 for both time points, Fig 2B). Unexpectedly, a transient decrease in calyx number was also detected on the side contralateral to ototoxic exposure at D3 post-cUL (** p < 0.01 vs SHAM and *** p < 0.001 vs D30 cUL, Fig 2C). Unlike the utricle, the Scarpa’s ganglion on the side ipsilateral to ototoxic exposure remains intact after chemical unilateral labyrinthectomy (Fig 3) with cell bodies without swelling, regardless of the time after this ototoxic exposure. In addition, representative Myo7a immunostaining of contralateral cochlea confirmed the preservation of hair cells, supporting the absence of detectable contralateral cochlear damage following cUL (S1 Fig). In conclusion, cUL induced a complete and permanent loss of type I hair cells afferents in the utricle exposed to p-arsanilic acid, accompanied by adaptive plasticity in the contralateral utricle relative to ototoxic exposure, while Scarpa’s ganglion remained unaffected.

### Chemical unilateral labyrinthectomy alters cochlea structure and auditory function on the side ipsilateral to ototoxic exposure

**Fig 4:**
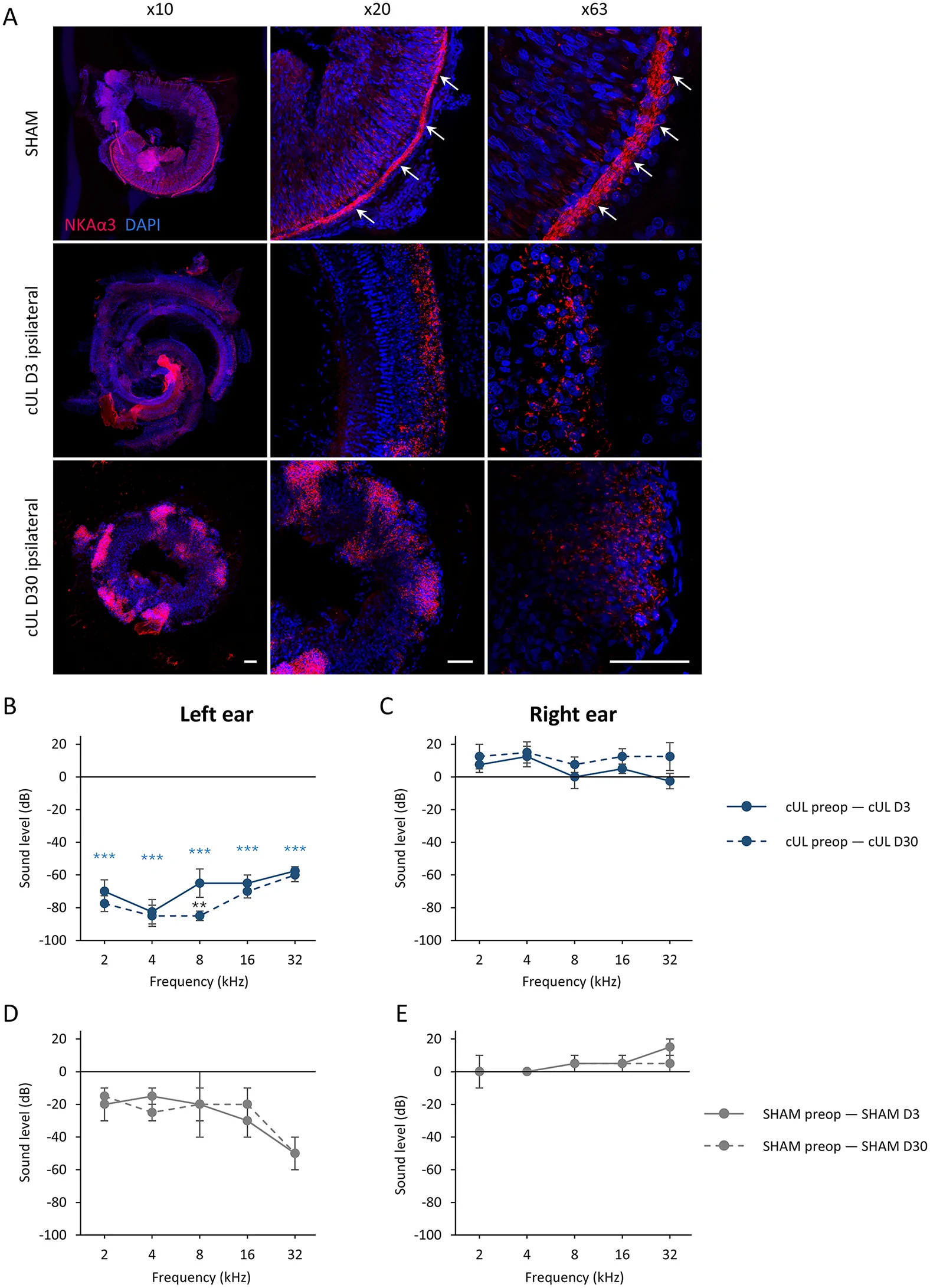
Chemical unilateral labyrinthectomy alters the cochlea structure and audition solely on the side ipsilateral to ototoxic exposure. Orthogonal maximum intensity projection of confocal immunostaining images of the cochlea on the side ipsilateral to ototoxic exposure (left), showing NKAα3+ (red) labeling of hair cells afferents and DAPI (blue) staining, in SHAM group (n = 5), three days (D3 cUL, n = 4) and thirty days (D30 cUL, n = 4) after cUL. Arrows indicate the thin layer of hair cells afferents present in the SHAM group and absent in the cUL group in acute (D3) or compensated (D30) delays. Scale bar = 50 µm. Curves indicate the mean auditory brainstem responses (ABR) threshold shifts in the ipsilateral (left, B) and in the contralateral (right, C) ears relative to ototoxic exposure in cUL animals (n = 4), as well as in the ipsilateral (D) and contralateral (E) ears relative to the surgery in SHAM animals (n = 2). Error bars represent SEM. A significant difference from the pre-operative value is indicated by * in blue. A significant difference between cUL D3 and cUL D30 is indicated with * in black. Statistical significance was only assessed in the cUL group using two-way ANOVA followed by Tukey’s multiple comparison post hoc test (** p < 0.01, *** p < 0.001).

An overview of the cochlea (x10), along with magnified views at x20 and x63, is presented in Fig 4A. Structural damage to the cochlea is observed from D3 and persists at D30, on the side ipsilateral to ototoxic exposure. These alterations affect hair cell afferents (NKAα3+) and nuclei (DAPI) compared with the SHAM group. Although no cell quantification or formal scoring was performed for this descriptive observation, the ipsilateral cochlea consistently appears altered relative to SHAM group. In contrast, the side contralateral to ototoxic exposure remains intact at both D3 cUL and D30 cUL, similar to the SHAM group (S2 Fig). Auditory brainstem responses (ABR) were assessed preoperatively and at 3 and 30 days after the surgery in cUL (n = 4) and SHAM (n = 2) groups (S3 Fig). Threshold shift analysis revealed a severe hearing loss in the ipsilateral ear (left) relative to ototoxic exposure in cUL animals at both postoperative time points (*** p < 0.001 vs. preoperative ABR threshold; Fig 4B), with an average loss of approximately 70 dB. No significant change in auditory thresholds was detected in the contralateral ear (right) in cUL animals relative to ototoxic exposure (Fig 4C). In the SHAM group, tympanic membrane perforation appeared to induce a mild hearing loss of about 30 dB, restricted to the left ear (Fig 4D and 4E).

### Chemical unilateral labyrinthectomy induces central medial vestibular nuclei plasticity

Unilateral vestibular loss induces numerous plastic events in the vestibular nuclei [46]. Among these events are cell proliferation, glial reaction, and neural KCC2 modulation, which are all involved in vestibular compensation [23,42,47]. We evaluated cell proliferation with a single injection of BrdU (200 mg/kg) 3 h prior to intracardiac perfusion at D3 post-cUL (S4A Fig). Only about 2.95 ± 0.8 BrdU+ cells are found in the MVN (S4B Fig) compared to about 20 BrdU+ cells after unilateral vestibular neurectomy [36,48,49]. In our chemical labyrinthectomy model, no survival hair cells were found at D30 post-cUL (n.s vs SHAM group).

Reduced KCC2 expression at the neuronal membrane reflects elevated intracellular Cl- ion concentration, weakening GABAergic inhibition and potentially shifting GABAergic signaling to excitatory [50]. In our model, there is no decrease in KCC2 membrane expression (S5A-B Figs). Unlike in the unilateral vestibular neurectomy, few cell proliferations, no newly generated survival cells, nor any neuronal KCC2 modulation have been found in this cUL model.

**Fig 5:**
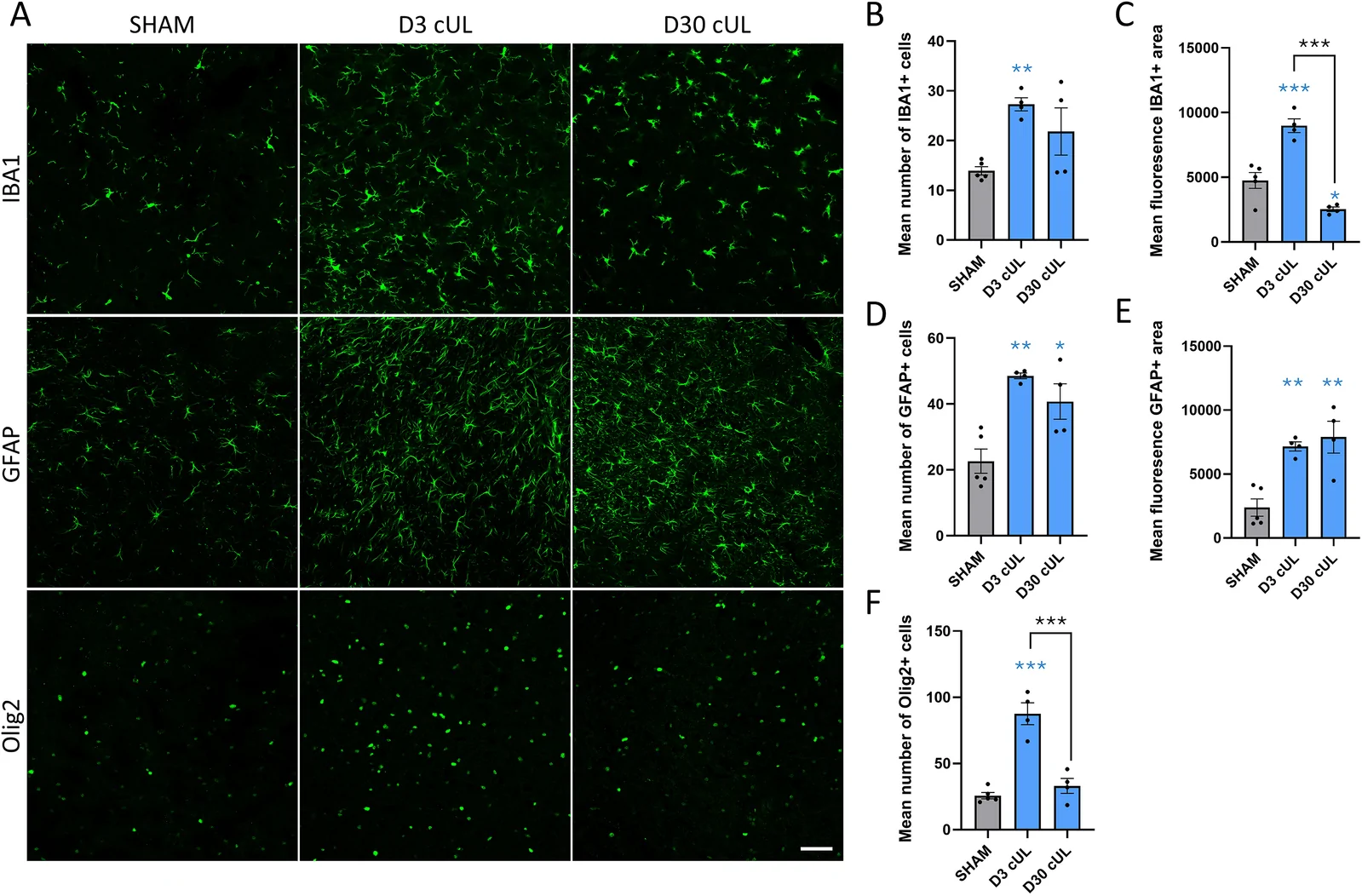
Chemical unilateral labyrinthectomy modulates glial reaction in the deafferented medial vestibular nucleus. A. Confocal immunostaining images of IBA1+ , GFAP+ and Olig2+ cells (green) in the deafferented medial vestibular nucleus (MVN) for SHAM group (n = 5), three days (D3 cUL, n = 4) and thirty days (D30 cUL, n = 4) after UL. Scale bar = 50 µm. Quantitative assessment of the cUL effect on the number of IBA1+ (B), GFAP+ (D), Olig2+ (F) cells in the deafferented MVN of SHAM (grey), D3 cUL (blue) and D30 cUL (blue) groups. Quantitative assessment of the cUL effect on the fluorescence IBA1+ (C) and GFAP+ (E) area in the deafferented MVN of SHAM (grey), D3 cUL (blue) and D30 cUL (blue) groups. The histogram represents group means ± SEM, and each point corresponds to the mean number of cells per animal analyzed. A significant difference from the SHAM group is indicated by * in blue for D3 cUL and D30 cUL groups. A significant difference between the D3 cUL and D30 cUL group is indicated by * in black (* p < 0.05, ** p < 0.01, *** p < 0.001; nested one-way ANOVA and Tukey’s post hoc test).

cUL induced glia cell plasticity in the MVN (Fig 5A) with a significant increase in microglia (IBA1; F_(2,10)_ = 7.146; ** p < 0.01 vs SHAM group), astrocytes (GFAP; F_(2,10)_ = 13.47; ** p < 0.01 vs SHAM group) and oligodendrocytes (Olig2; F_(2,10)_ = 35.96; *** p < 0.001 vs SHAM group) at D3 post-cUL in the MVN (Figs 5B, 5D and 5F). These results are consistent with those previously described in UVN model [24,36]. At D30 post-cUL, only a significant increase in astrocytes persists (F_(2,10)_ = 13.47; * p < 0.05 vs SHAM group) although microglial number remains high and shows no difference with D3 cUL group. cUL might induce morphological changes in microglial cells (Fig 5C). The fluorescence IBA1 + area increases at D3 cUL (F_(2,10)_ = 45.03; *** p < 0.001 vs SHAM group) and decreases at D30 cUL (F_(2,10)_ = 45.03; * p < 0.05 vs SHAM group). Since the number of IBA1 + cells in D30 cUL is similar to that in SHAM with a tendency toward an increase in microglia, the marked decrease in fluorescence might reflect a smaller immunoreactive area and an amoeboid morphology of these cells. However, consistent with an increased number of GFAP+ cells in the MVN at D3 and D30 after cUL, the fluorescence GFAP+ area (Fig 5E) increases as well compared to SHAM group (F_(2,10)_ = 15.41; ** p < 0.01).

### Chemical unilateral labyrinthectomy induces typical vestibular disorders

**Fig 6:**
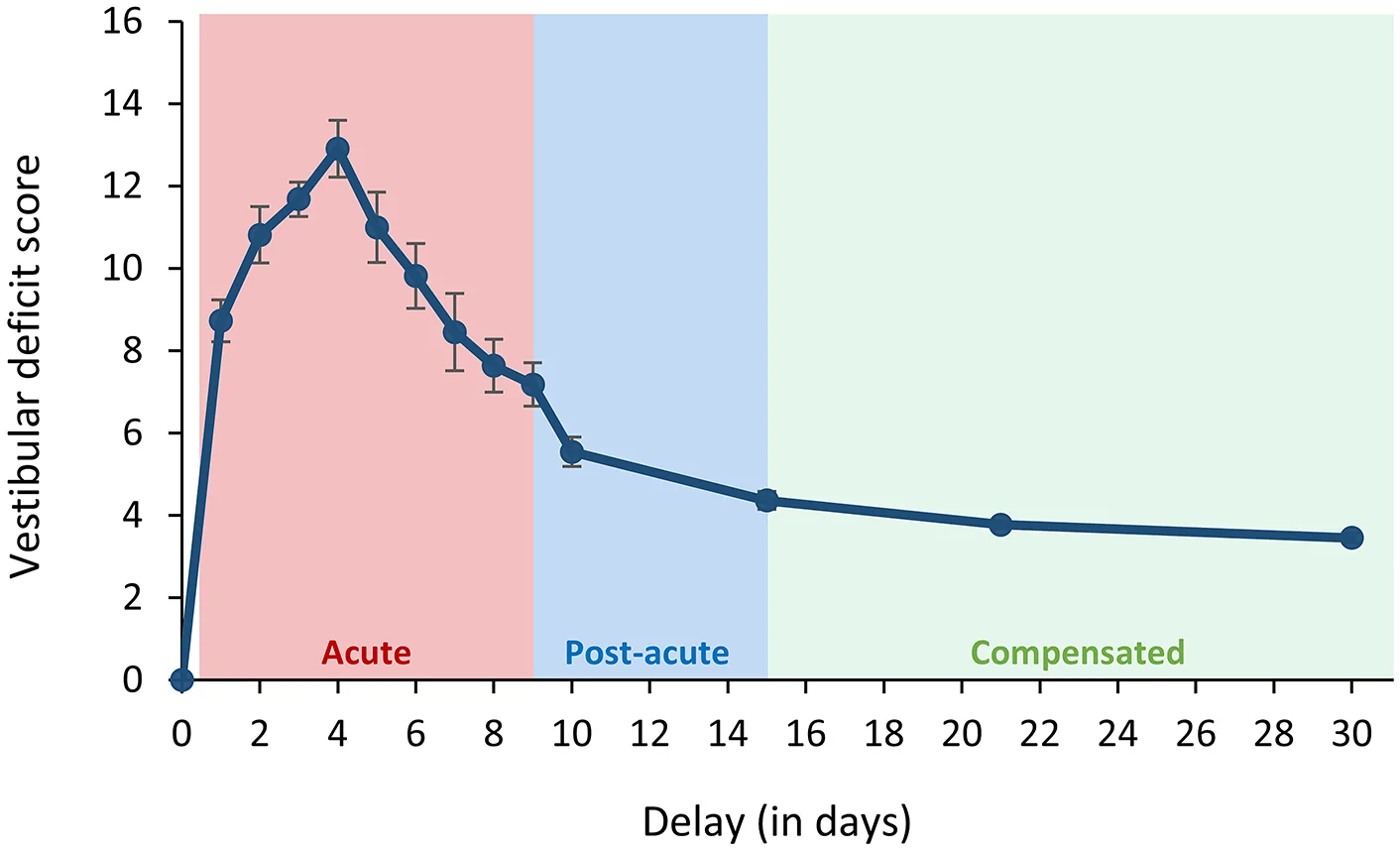
Qualitative assessment of vestibular syndrome. Curve illustrating the time course of the vestibular syndrome of the cUL group divided into three phases: acute (after surgery to D9), post-acute (D9 to D15) and compensated (D15 to D30).

The behavioral deficits were assessed at several time points over a period of 30 days using a qualitative vestibular score (see Methods section 5.5.1.). Vestibular syndrome was observed as early as D1 post-cUL (8.73 ± 0.5), with a peak at D4 (12.9 ± 0.69), reflecting the progressive development of the ototoxic lesion induced by cUL (Fig 6). Thereafter, the vestibular deficits decreased through two distinct phases. Until D9, during the acute phase, vestibular symptoms remained pronounced, with only a limited reduction in syndrome severity despite the onset of functional recovery. Then, in the post-acute phase, a progressive and linear reduction in vestibular score was observed, reaching a value of 4.36 ± 0.21 at D15 (Fig 6). In the compensated phase, from D15 to D30, the vestibular score reached a plateau, and inter-individual variability decreased, reflecting more similarities in the behavioral patterns across animals. However, the syndrome did not completely disappear 30 days after the cUL (3.45 ± 0.13), with residual signs such as head tilt, bobbing, tail-hanging reflex and locomotion impairment, while barrel rolling and circling behaviors were no longer observed.

### Chemical unilateral labyrinthectomy induces acute and persistent postural deficit

**Fig 7:**
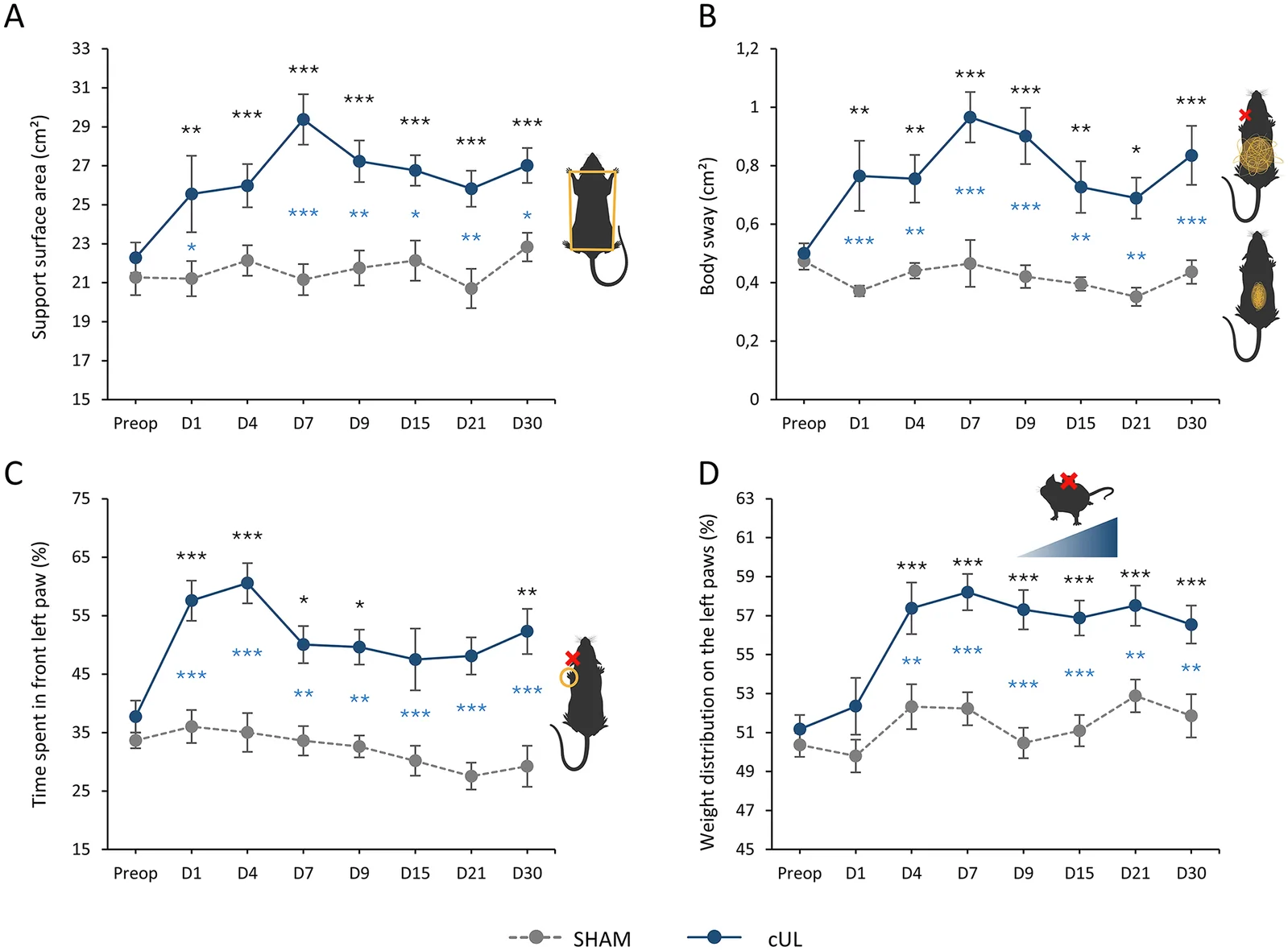
Effect of the chemical unilateral labyrinthectomy on static postural parameters. Curves indicating the mean post-operative recovery of the support surface area (A), the body sway (B), the time spent in the front left paw (C) and the weight distribution on the lateral axis (D) of SHAM (n = 12) and cUL (n = 11) groups. Error bars represent SEM. A significant difference from the pre-operative value is indicated by * in black for cUL group. A significant difference between SHAM and cUL group is indicated with * in blue (* p < 0.05, ** p < 0.01, *** p < 0.001; repeated measure two-way ANOVA and Sidak’s post hoc for statistical differences between the two groups and Dunnett’s multiple comparison post hoc test to compare values at each postoperative time with the preoperative value).

The support surface area parameter, delimited by the animal’s four paws, is commonly used to describe postural stability and restoration of balance [33,41,51]. The SHAM group maintained a stable support surface area throughout the entire period (21.65 ± 0.88 cm²; no delay effect). For the cUL group, the support surface area was significantly increased as soon as the first post-cUL day (25.55 ± 1.96 cm², Time x Group: F_(7, 147)_ = 5.215; * p < 0.05 vs SHAM group) and did not return to pre-operative values at D30 (27.02 ± 0.89 cm², Time x Group: F_(7, 147)_ = 5.215; *** p < 0.001 delay effect; Fig 7A).

The body sway, an index of instability commonly used in clinical posturology, quantifies the barycenter dispersion while the animal is static and on all four paws (Fig 7B). The SHAM group maintained a stable support surface area throughout the entire period (0.42 ± 0.034 cm²; no delay effect). For the cUL group, the body sway was significantly increased as soon as the first post-cUL day (0.77 ± 0.12 cm², Time x Group: F_(7, 147)_ = 4.607; *** p < 0.001 vs SHAM group) and persisted at D30 (0.84 ± 0.10 cm², Time x Group: F_(7, 147)_ = 4.607; *** p < 0.001 vs SHAM group and delay effect).

SHAM group consistently spent approximately 32.24 ± 2.53% of the time on their front left paw during the 5-minute acquisition regardless of the delay (no delay effect, Fig 7C). In contrast, cUL animals showed increased and sustained use of their front left (ipsilateral side relative to ototoxic exposure) paw from the first post-cUL day to D30 post-cUL (Time x Group: F_(7, 147)_ = 2.748; *** p < 0.001 vs SHAM group at both time points).

We then quantified the percentage of weight distribution along the lateral body axis, between the left and right paws (Fig 7D). SHAM group distributed their weight equally between the left and right paws regardless of the delay (51.38 ± 0.87% vs 48.62 ± 0.87%; no delay effect). Prior to the ototoxic exposure, cUL rats exhibited a balanced weight distribution between the left and right paws (51.18 ± 0.72% vs 48.82 ± 0.72%). These results are consistent with those previously described in UVN model [33,37]. From D4 to D30 post-cUL, cUL rats exhibited a significant increase in their weight applied on the left (ipsilateral side relative to ototoxic exposure) paw (about 57.31 ± 1.03%; Time x Group: F_(7, 147)_ = 2.929; ** p < 0.01 at D4, D21, D30 and *** p < 0.001 at D7-D15 vs SHAM group). In conclusion, cUL induced postural instability and both temporal and weight distribution biases toward the side ipsilateral to ototoxic exposure.

**Fig 8:**
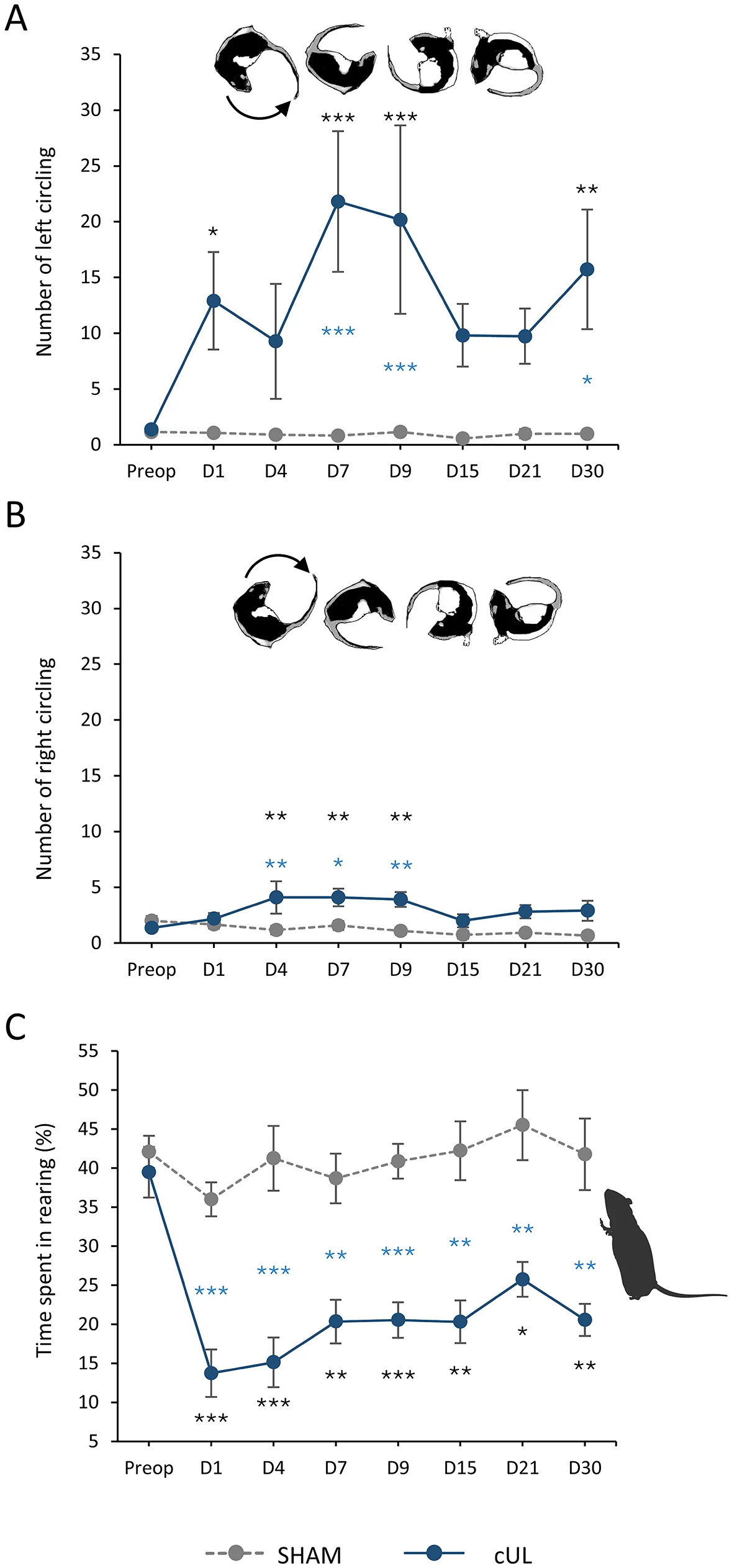
Effect of the chemical unilateral labyrinthectomy on dynamic postural parameters. Curves indicating the mean post-operative recovery of the number of left (A) and right (B) circling, and the time spent rearing (C) of SHAM (n = 12) and cUL (n = 11) groups. Error bars represent SEM. A significant difference from the pre-operative value is indicated by * in black for cUL group. A significant difference between SHAM and cUL group is indicated with * in blue (* p < 0.05, ** p < 0.01, *** p < 0.001; repeated measure two-way ANOVA and Sidak’s post hoc for statistical differences between the two groups and Dunnett’s multiple comparison post hoc test to compare values at each post-operative time with the pre-operative value. Sphericity Greenhouse-Geisser correction was applied to the time spent in rearing).

### Chemical unilateral labyrinthectomy induces acute and persistent dynamic deficit

Before surgery, none of the two groups performed left-hand circling (1.17 ± 0.24 for SHAM group and 1.37 ± 0.2 for D30 cUL group; Fig 8A). The SHAM group did not perform left nor right circling after surgery: the number of turns per acquisition varied very little from the preoperative session to the D30 delay. For the cUL group, the number of left fast laps per session increased from D1 post-cUL (12.01 ± 4.36; Time x Group: F_(7, 147)_ = 3.096; * p < 0.05 delay effect) to D30 post-cUL (15.73 ± 5.37; Time x Group: F_(7, 147)_ = 3.096; ** p < 0.01 delay effect) with a peak a D7 (21.82 ± 6.30; Time x Group: F_(7, 147)_ = 3.096; *** p < 0.001 vs SHAM group and delay effect). At the same delay, the cUL group exhibit only about 4.09 ± 0.79 right circling (Fig 8B).

The SHAM group spent about 41.06 ± 3.33% of their time in rearing regardless of the delay after the surgery. In contrast, the cUL group spent only 13.74 ± 3.03% (Time x Group: F_(7, 147)_ = 3.624; *** p < 0.001 vs SHAM group and delay effect, Fig 8C) of their time in rearing, indicating an important instability of the animal in D1 post-cUL. This instability remains at D30 post-cUL (Time x Group: F_(7, 147)_ = 3.624; ** p < 0.01 vs SHAM group and delay effect).

### Chemical unilateral labyrinthectomy induces acute and persistent anxiety-like behavior

**Fig 9:**
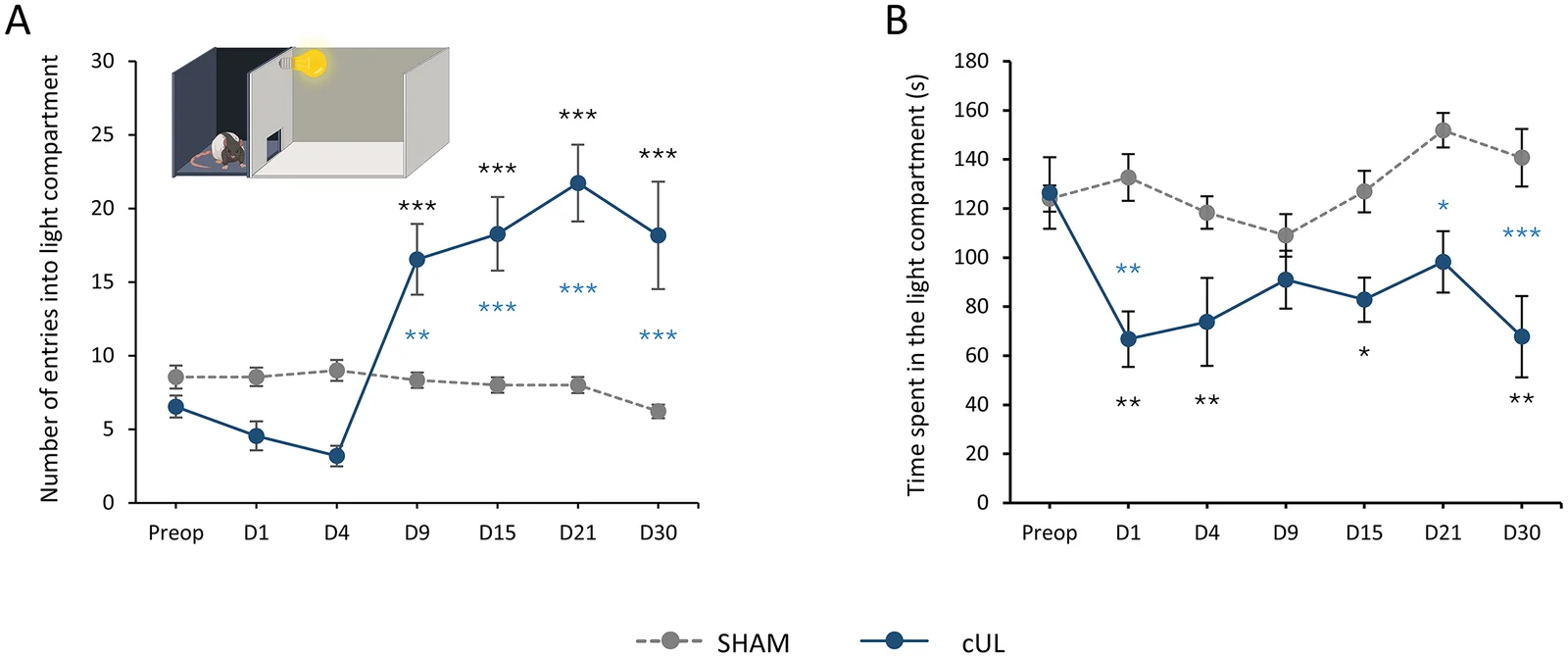
Effect of the chemical unilateral labyrinthectomy on anxiety-like behavior. Curves indicating the mean post-operative recovery of the number of entries into the light compartment (A) and the time spent in the light compartment (B) in the light/dark box test of SHAM (n = 12) and cUL (n = 11) groups. Error bars represent SEM. A significant difference from the pre-operative value is indicated by * in black for cUL group. A significant difference between SHAM and cUL group is indicated with * in blue (* p < 0.05, ** p < 0.01, *** p < 0.001; repeated measure two-way ANOVA and Sidak’s post hoc for statistical differences between the two groups and Dunnett’s multiple comparison post hoc test to compare values at each post-operative time with the pre-operative value).

The LDBT can be used to assess the animals’ level of anxiety-like behavior, as the light zone is naturally avoided by rodents. First, we quantified the number of entries into the light compartment during the whole 5-minutes acquisition (Fig 9A). The SHAM group did approximatively 8.1 ± 0.6 entries into the light compartment regardless of the delay (no delay effect). In contrast, the cUL group first decreased the number of entries from D1 to D4 post-cUL (minimum at 3.18 ± 0.7 entries; n.s. vs SHAM group). This slight decrease can be explained by the increased immobility of the animals in this test because of the vestibular syndrome. Then, the number of entries significantly increased from D9 (16.55 ± 2.4; Time x Group: F_(6, 108)_ = 11.88; ** p < 0.01 vs SHAM group) to D30 post-cUL (18.18 ± 3.65; Time x Group: F_(6, 108)_ = 11.88; *** p < 0.001 vs SHAM group), reflecting the hyperexcitability of injured animals. As for the time spent in the light compartment, the SHAM group maintained a high level of exploration throughout the study with approximately 129.06 ± 8.23 seconds (Fig 9B). In contrast, the cUL group spent significantly less time in the light compartment in acute phase (D1, 66.73 ± 11.24 seconds; Time x Group: F_(6, 108)_ = 2.574; ** p < 0.01 vs SHAM group) and compensated phase (D30, 67.73 ± 16.56 seconds; Time x Group: F_(6, 108)_ = 2.574; *** p < 0.001 vs SHAM group) demonstrating anxiety-like behavior induced by the cUL.

## Discussion

This study highlights the cellular plasticity events in the peripheral and central vestibular systems following a chemical unilateral labyrinthectomy (cUL) induced by p-arsanilic acid in rodents. In our study, we show peripheral plasticity in the utricle contralateral to the ototoxic exposure, as well as central plasticity in the ipsilateral VN. Additionally, behavioral parameters (posture, hyperactivity and anxiety) are altered early on from the first day following this ototoxic exposure and persist over an extended period (up to 30 days).

### Chemical unilateral labyrinthectomy alters the inner ear

#### Chemical unilateral labyrinthectomy alters cochlea and audition solely on the side of the ototoxic exposure.

In the present cUL model, p-arsanilic acid, an ototoxic compound, is introduced into the middle ear after perforation of the tympanic membrane. Considering the inner ear’s anatomy, it is likely that our compound diffuses through the oval window (to the vestibule) and round window (to the cochlea). Previous studies have shown that substances injected into the ear can reach both the vestibular and cochlear epithelia, particularly via the round window membrane [52,53]. Additionally, this membrane’s permeability is increased by nearby suction [54], an approach integrated in our protocol, which combines instillation and aspiration of p-arsanilic acid. Taken together, these elements may explain the cochlear alterations observed by immunofluorescence. We suspect that p-arsanilic acid disturbed the cochlea hair cells or supporting cells layout. Although our findings differ from those of [20], who found no damage to the cochlear hair cells, it is important to note that they used hematoxylin-eosin staining on sections, whereas we employed whole-mount immunofluorescence, which may reveal subtler alterations. Consistently, ABR assessments demonstrated a profound hearing loss in cUL animals on the side of the ototoxic exposure, confirming auditory impairment following arsanilic acid ototoxic exposure [55]. As seen in other vestibular lesion models [9], these deficits were strictly confined to the ear exposed to ototoxic compound. Our results indicate that arsanilic acid is not restricted to the vestibular structures and impacts both cochlear integrity and hearing. Although both systems were similarly impacted on the side exposed to the ototoxic compound, peripheral plasticity occurred solely in the vestibular system contralateral to this exposure. This vestibular-specific adaptation will be addressed in the next section.

#### Chemical unilateral labyrinthectomy induces contralateral peripheral vestibular plasticity.

We did not find any alteration of the primary neurons of Scarpa’s ganglion in the side exposed to the ototoxic compound, as in these previous study [20,22]. Furthermore, Zwergal et al., using the same model as us, detected inflammation in the vestibular nerve using in vivo PET scanning [10]. This aspect reinforces the link between our model and acute peripheral vestibular disorders, as it is the vestibular nerve rather than the vestibular ganglion cells that show signs of inflammation [12,56].

Our result shed light into a complete and irreversible loss of calyx around type I hair cells in the ipsilateral utricle subjected to p-arsanilic acid ototoxic exposure. Calyx retraction precede type I hair cell extrusion and their loss within the vestibular epithelium [57,58]. As calyx retraction observed in our model cannot directly be assimilated to type I hair cell loss [59], the nuclei shape in the utricle subjected to the ototoxic exposure strongly suppose a complete loss of type I hair cells, agreeing with previous reports indicating a complete vestibular hair cell loss after p-arsanilic exposure [20,22,60–63]. The toxicity of p-arsanilic acid likely alters the endolymphatic environment [61], leading to vacuole formation in the sensory epithelia (utricle, saccule and ampullar) as early as one day after the ototoxic exposure, and remaining visible up to one month [20,22]. In addition, three months after p-arsanilic application, a restructuration of a non-sensory epithelial monolayer occurs [22]. This indicates a complete and irreversible destruction of both type I and type II hair cells using p-arsanilic acid. Other ototoxic compounds have been shown to damage hair cells, and even after eight months post-injury, regenerated cells fail to recover the normal number and morphology of native hair cells [64,65]. As hair cell regeneration originate from supporting cells [66–70], the limited regenerative outcome suggests either direct damage to these cells or impairment of their regenerative ability via the ototoxic compound [71,72]. This may also apply to our model using p-arsanilic acid, supporting the idea of irreversible hair cell damage on the side exposed to this compound.

Unexpectedly, at D3 post-cUL, we observed a transient decrease in the number of calyx afferents innervating type I hair cells on the side contralateral to the ototoxic exposure, which returned to control levels by D30. Several lines of evidence argue against p-arsanilic diffusion in our model. Prolonged exposure without compound removal did not produce any extra-ipsilateral effects [22] and our protocol further minimizes this risk by limiting exposure to 4 minutes. Moreover, because p-arsanilic acid induces irreversible hair-cell toxicity [20,22,60–63], any contralateral diffusion should result in bilateral vestibular deficits, which are not observed in our cUL model. Consistently, cochlear damage and hearing loss remain strictly ipsilateral to ototoxic exposure. This finding therefore suggests a peripheral adaptive response triggered by central mechanisms, and from a speculative point of view, could involve the efferent vestibular system. Following unilateral vestibular damage, electrophysiological imbalance between both VN occurs, with increased excitability observed in the non-deafferented VN [73–75]. This central imbalance could be detected by the efferent vestibular system which receives afferences from both medial vestibular nuclei (MVN) [76]. In addition, the ipsilateral efferent vestibular nuclei mainly connects the contralateral vestibular hair cells (type I and II) [77–80]. By directly reducing vestibular hair cells sensitivity [81], the efferent system could transiently decrease the excitability of contralateral afferents to counterbalance the central asymmetry induced by cUL. Supporting cells may reinforce this effect by modulating synaptic density [82–85]. The recovery of calyx number by D30 post-cUL suggests that this peripheral modulation fades once spontaneous activity of central vestibular neuron is restored [86,87]. A transient loss of contralateral calyces likely reflects synaptic remodeling rather than hair-cell loss followed by hair cell proliferation. In adult mammals, hair-cell regeneration is rare and delayed by weeks [64,68,88], whereas calyces exhibit rapid structural plasticity, with a reversible retraction reported after sub-chronic ototoxic exposure [57,58]. This dynamic fits well with the transient decrease in calyx number observed at D3 post-cUL, since calyx retracts within 24 h and reforms within days after excitotoxic damage [27]. Taken together, these findings highlight a surprising adaptive reactive plasticity of the contralateral utricle that was not exposed to ototoxic compound that could contribute to vestibular compensation. To deepen our understanding, future research should focus on an ultrastructural analysis by electron to assess the complete morphological restoration of type I hair cell calyces microscopy [27], as well as the study of synaptic contact at vestibular hair cells.

### Chemical unilateral labyrinthectomy induces deafferented medial vestibular nuclei plasticity

Our cUL model induced few cell proliferation but no survival, suggesting that this reactional plasticity is specific to vestibular nerve section models [24,89]. This difference may stem from the greater severity of nerve section [90], including loss of the Scarpa’s ganglion [7,89,91]. Other responses, such as potassium-chloride co-transporter KCC2 downregulation in neurons, also seem restricted to nerve section models [42,49]. Our results contrast with those of [92] who reported BrdU+ cells after p-arsanilic acid exposure, but their multiple BrdU injections between D11–D15 likely captured a broader window than our single BrdU injection during the critical period, potentially explaining the discrepancy.

Following unilateral vestibular deafferentation, restoring a level of homeostatic excitability within VN is essential for functional recovery. In the cUL model, this central compensation may rely more on pre-existing neuronal or glial cells than on the generation of new cells. Indeed neuroglia (astrocyte, microglia and oligodendrocytes) are key players responsible for the homeostatic support of the central nervous system [93] and are upregulated in other vestibular models [4,36,94]. Moreover, glial reactivity has been reported in vivo and ex vivo in the deafferented VN and might accelerate vestibular compensation [10]. Our focus will be limited to the deafferented side, given that glial plasticity has been consistently reported to occur only in the deafferented VN [10,49,94].

Astrogliosis, characterized by increased GFAP expression [95,96], was observed in our model at both acute and compensated stages, consistent with typical astrocyte reactivity following injury. Since few cell proliferations has been observed in our model, increased number of GFAP+ cells might result from astrocyte migration or upregulation of GFAP expression in astrocytes that do not express detectable levels of GFAP in sham group. This phenomenon has been previously reported in cerebral pathologies [97]. Beyond this reactive profile, astrocytes are known to support synaptic plasticity and neural network reorganization in the central nervous system [98,99]. In particular, a specific astrocytic subtype emerged in the deafferented MVN, potentially contributing to synaptogenesis [100]. In addition, astrocytes can modulate glutamatergic transmission [101] which is a critical driver for vestibular compensation [102]. As vestibular recovery involves sensorimotor relearning [103], astrocytes, as key component of the tripartite synapse, likely play a pivotal role in the central plastic changes [104], necessary for compensation. Taken together, these findings highlight astrocytes as central regulators of neuronal excitability, positioning them as key mediators of the adaptive plasticity underlying vestibular compensation.

Microglia in reactive states increase IBA1 expression [105], as observed in our model only in acute phase of the vestibular syndrome. Microglia are known to regulate neuronal excitability, by reducing the activity of overly excited neurons, most likely through astrocytes [106,107]. This raises the question of whether they could increase neuronal activity and thus restore excitability in the deafferented VN through other mechanisms. Furthermore, microglia support myelin repair processes by facilitating oligodendrocyte progenitor proliferation and differentiation [108]. Morphologically, homeostatic microglia exhibits a ramified shape, whereas reactive microglia is often associated with hypertrophic forms [109–111]. In our model, microglia morphology seems to differ between acute and compensated phases with amoeboid or deramified form at D30. Since microglia can be protective during the acute phase of injury but become detrimental if reactivity persists, further morphological profiling, such as through Sholl analysis, could provide deeper insights into their impact across vestibular recovery stages.

Glial reactivity occurs in both the vestibular nerve and the VN in our model, suggesting a reactive inflammation [10] that could initiate the generation of new oligodendrocytes [112]. Our cUL model induced an increase in Olig2 + cells only at D3, returning to control values at D30, in line with previous findings following cortical brain injury [113]. After brain damage, increased Olig2 + cells numbers at the lesion site can result from Olig2 transcriptional up-regulation, cell proliferation or cell migration [113]. In our cUL model, we did not detect any cell proliferation in the VN, whereas others brain injury models [113] or vestibular models did [36]. This suggests that Olig2 + cell increase in our model may result from transcriptional regulation or migration. As oligodendrocytes are essential for preserving myelin integrity, their dysfunction may worsen signal transmission and delay recovery [114]. Unilateral vestibular lesions lead to decreased activity within the VN. Sensory substitution through visual and proprioceptive inputs—known to modulate VN activity [115,116]— plays a crucial role in compensation [18]. Increased activity in these sensory pathways could be reinforced by adaptive myelination [117] and could facilitate more efficient signal transmission to restore or maintain VN activity. Therefore, oligodendrocytes might reinforce substitutional input that would be beneficial to vestibular compensation. Another element to consider is that Olig2 marks oligodendrocyte progenitor cells (OPC), as well as mature oligodendrocytes [118] and that this transcription factor is plastic [119,120]. Thus, our transient increase in Olig2 + could also come from OPC migration or from OPC differentiation into oligodendrocyte. Also, an Olig2+ increase has been reported in response to stress tissue where it can be involved in oligodendrocyte lineage stabilization and cell survival [121,122]. Thus, our cUL model could induce a transient stress tissue that disappears once most of vestibular deficits are compensated.

Electrophysiological imbalance between both VN in the acute phase of vestibular syndrome may place the central nervous system in a state of functional disruption, thereby triggering rapid compensatory mechanisms involving widespread glial reactivity to restore homeostasis. In the compensated phase, however, only a strong astroglial response would persist and be sufficient to stabilize neuronal networks and maintain this electrophysiological balance that underlies vestibular compensation.

### Chemical unilateral labyrinthectomy alters postural parameters and anxiety-like behavior

cUL leads to damage of vestibular hair cells, and their loss correlates with impaired vestibular function [66,123]. Consistent with our findings, other studies have reported vestibular dysfunction (such as tail hanging and landing reflexes and head tilt) following p-arsanilic acid exposure [21]. In our study, we defined three phases of the vestibular syndrome: acute, post-acute, and then compensated. Among the various qualitative assessment described in the literature, this vestibular syndrome kinetics induced by p-arsanilic acid remains similar, with symptoms peaking a few days after ototoxic exposure and plateauing one month after this ototoxic exposure [18–20,22]. Our model contrasts sharply with the peak observed at D1 in models involving vestibular nerve section [24,33,36,48,49]. As discussed previously, this difference in kinetics may be due to the sudden and complete disruption caused by surgical section [7], whereas our model induces a more gradual alteration of the vestibular sensory organs. This gradual degeneration in vestibular hair cells may account for the delayed onset of vestibular symptoms in our model, which peak a few days after the ototoxic exposure and persist for up to a month. The long-lasting nature of these deficits likely reflects permanent hair cell loss on the side exposed to the ototoxic compound. Despite adaptative plasticity and the normalization of contralateral calyces’ number, such compensatory mechanisms may not be sufficient to fully compensate for vestibular deficits induced by cUL.

Static postural deficits following a vestibular lesion are thought to result from the electrophysiological imbalance between the VN, whereas dynamic vestibular compensation mainly relies on sensory substitution mechanisms [124]. In our model, the support surface area was significantly enlarged from the first post-cUL day and remained above preoperative values at D30, as previously reported [33,51]. Interestingly, while the peak in support surface area occurred at D3 in nerve section models, it was delayed until D7 in our cUL model, which aligns with a more gradual degradation of vestibular sensory inputs. Postural instability is observed immediately after the ototoxic exposure, as evidenced by a sustained increase in body sway over time. In response, the animal adopts an adaptive postural strategy characterized by a shift in weight distribution toward the side ipsilateral to ototoxic exposure—however, this shift does not fully resolve the instability. This postural bias appears as early as D4, contrasting with nerve section models where it typically emerges later, between D7 and D10 [33,36,37,48], suggesting that such postural strategy is conserved across various vestibular deafferentation models. As more weight is applied to the ipsilateral paws relative to ototoxic exposure, the center of gravity is displaced toward the deafferented side, potentially accounting for the increased use of the ipsilateral forepaw as a stabilizing anchor. However, the weight distribution shift strategy appears maladaptive, as it is abolished by pharmacological treatments [36,49] or rehabilitation protocols [48] that promote vestibular compensation.

Circling behavior, a rapid rotational behavior linked to cerebral asymmetry, is frequently observed in rodent models of neurological disorders such as Parkinson’s disease, anxiety and vestibular impairments [5,33,125–127]. A similar behavior has also been reported in humans [128,129]. In our study, circling did not return to preoperative values at D30, despite the restoration of electrophysiological balance between both VN. This persistence may reflect the onset of hyperactivity induced by vestibular deafferentation, typically emerging after the acute phase (from D9 onward). As for the nerve section model [130], we also observe a hyperactivity phase at the same delay during our LDBT assessment with frenetic back and forth between both compartments. Several hypotheses may account for the hyperactivity observed during the compensated phase. This behavior could be a compensatory mechanism to compensate for the lack of exploration in animals with vestibular damage during the acute phase, which results in suboptimal arousal levels and reduced sensory feedback [131]. Increasing walking speed may also serve as a compensatory strategy to stabilize balance, as reported in both rodents and humans [130,132]. In our model, animal display immobility in LDBT during the acute phase, consistent with previous findings using Ethovision® [18,130]. The absence of early hyperactivity and predominantly static posture during the acute phase of the vestibular syndrome may represent an avoidance strategy to fall, similar to that observed in humans with vestibular disorders [132]. Additionally, peripheral vestibular loss may not be the only trigger for locomotor hyperactivity which may emerge through long lasting complex changes in the basal ganglion and motor associated regions that takes time [60].

Regarding anxiety-like behavior, animals consistently spent less time in the light compartment across all phases of vestibular compensation. During the acute stage, this may reflect their predominantly static posture where the animals favored a quadruped posture. In contrast, during the compensated phase, despite exhibiting hyperactivity behavior and increased velocity, animal still avoided the light compartment, suggesting persistent anxiety-like behavior, as previously reported [39,133].

### Clinical relevance and limitations

Signs of inflammation of the vestibular nerve, but not of Scarpa’s ganglion, have been described in patients with acute unilateral vestibulopathy [12,56], and are replicated in our cUL model [10,22]. Acute inflammation may initially be beneficial by promoting tissue repair through tightly regulated cytokine signaling [134–136]. In contrast, excessive or prolonged glial activation can lead to chronic neuroinflammation associated with persistent tissue alterations and degenerative processes [134,135]. It is therefore plausible that chronic inflammation may be present in patients who fail to recover or compensate, raising questions about whether glial reactivity is tightly regulated and whether it could be therapeutically targeted, potentially in a patient-specific manner. In acute unilateral vestibulopathy, elevated levels of tumor necrosis factor α (TNF-α) and C-reactive protein (CRP) have also been reported [137,138]. However, these findings are not consistently observed across studies, leaving open questions about inflammation as a core feature of this disorder. This issue is clinically relevant, particularly when considering the potential use of anti-inflammatory treatments in acute unilateral vestibular disorders [56]. In this context, TSPO ligand imaging represents a promising approach to investigate glial reactivity in the vestibular nerve and nucleus of patients following acute unilateral vestibulopathy as an individualized biomarker, as has been established for patients with vestibular schwannoma [139,140].

We hypothesized that peripheral plasticity after cUL requires central detection of the lesion and may involve the efferent vestibular system (EVS) as a key modulator of bilateral peripheral vestibular plasticity. Mice with genetic alterations in cholinergic EVS signaling exhibit baseline posturo-locomotor deficits [141,142]. Moreover, EVS has been implicated in vestibular compensation of the vestibulo-ocular reflex following unilateral vestibular lesion [143–145]. Although the EVS remains poorly characterized in humans, recent reviews suggest a broader functional role than previously appreciated [146].

Finally, it should be noted that permanent unilateral hearing loss induced by cUL is inconsistent with the clinical definition of acute unilateral vestibulopathy in humans [147], which excludes hearing loss. This difference should be considered when extrapolating our findings the human condition. In addition, SHAM surgery induced moderate ipsilateral hearing loss due to tympanic membrane perforation. While this may contribute to some behavioral measures, it is unlikely to account for the pronounced vestibular deficits specifically observed after cUL.

## Conclusion

Recovery from unilateral vestibulopathy largely depends on central neuroplasticity, which is tightly regulated by neuroglial dynamics. Our findings highlight early and sustained astrocytic reactivity, transient microglial and oligodendrocyte responses, and peripheral plasticity within the utricle contralateral to ototoxic exposure. The contralateral plasticity highlighted in our cUL model suggests that, as with the non-deafferented VN, the non-exposed ear can no longer be considered a control. These central and peripheral changes mirror the clinical heterogeneity observed in patients, where persistent symptoms may arise despite apparent resolution of the acute phase of the vestibular syndrome. Unlike the vestibular nerve section model, which mimics a rare clinical condition, cUL provides a more representative model of common vestibular disorders. It offers a relevant platform to explore glia-targeted treatments and strategies promoting peripheral repair, especially for hair cell regeneration. Future studies should investigate glial cell modulation, either pharmacologically or optogenetically [148–150], to assess their contribution to vestibular compensation. Additionally, ultra structural morphological analysis of calyx and quantification of type I and type II hair cell population should be assessed along with supporting cells number in bilateral vestibular periphery since vestibular functional recovery relies on coordinated deafferented central and contralateral peripheral plasticity.

## Supporting information

S1 Fig Representative intact contralateral cochlea after chemical unilateral labyrinthectomy. Whole-cochlea view and high-magnification confocal images of the contralateral cochlea from a cUL animal, showing Myo7a (green) hair cells, NKAα3 (red) hair cell afferents, and DAPI (blue) nuclear counterstaining. Zoomed-in panel shows the region indicated by the white square in the whole-cochlea view. Inner hair cells (IHC) and outer hair cells (OHC) are indicated by brackets. Scale bars = 200 µm (whole cochlea) and 20 µm (zoomed panel).
(EPS)

S2 Fig Chemical unilateral labyrinthectomy specifically alters cochlear structure on the side ipsilateral to ototoxic exposure. Orthogonal maximum intensity projections of confocal immunostaining images of the cochlea on the ipsilateral (left) and contralateral (right) sides relative to ototoxic exposure, showing NKAα3+ (red) labeling of hair cells afferences and DAPI (blue) staining, in SHAM group (n = 5), three days (D3 cUL, n = 4) and thirty days (D30 cUL, n = 4) after cUL. Arrows indicate the thin layer of hair cells present in the SHAM group and right cochlea of both D3 and D30 cUL while it remains absent in the left cochlea of the acute (D3) or compensated (D30) delays in the cUL group. Scale bar = 50 µm.
(EPS)

S3 Fig Chemical unilateral labyrinthectomy alters auditory function on the side ipsilateral to ototoxic exposure. Curves indicate the mean auditory brainstem responses (ABR) threshold in the ipsilateral (left, A) and in the contralateral (right, B) ears relative to ototoxic exposure in cUL animals (n = 4), as well as in the ipsilateral (C) and contralateral (D) ears relative to the surgery in SHAM animals (n = 2). Error bars represent SEM. A significant difference from the pre-operative value is indicated by * in blue. A significant difference between cUL D3 and cUL D30 is indicated with * in black. Statistical significance was assessed only in the cUL group using two-way ANOVA followed by Tukey’s multiple comparisons post hoc test (** p < 0.01, *** p < 0.001). E. Representative ABR traces at 8 kHz for one cUL animal at the three time points with the left ear on top and right ear on the bottom. Blue arrows indicate the ABR threshold, defined as the lowest stimulus intensity that consistently evoked a reproducible waveform.
(EPS)

S4 Fig Chemical unilateral labyrinthectomy does not increase cell proliferation in the deafferented medial vestibular nucleus. A. Confocal immunostaining images of BrdU+ cells (red) in the deafferented medial vestibular nucleus (MVN) for SHAM group (n = 5), three days (D3 cUL, n = 4) and thirty days (D30 cUL, n = 4) after cUL. Scale bar = 50 µm. B. Quantitative assessment of the cUL effect on the number of BrdU+ cells in the deafferented MVN of SHAM (grey), D3 cUL (blue) and D30 cUL (blue) groups. SHAM and D3 cUL differed significantly, with mean values of 0.28 and 3.2 BrdU+ cells, respectively. The value “3” on the y-axis is marked with a red circle, indicating a low overall number. The histogram represents group means ± SEM, and each point corresponds to the mean number of cells per animal analyzed. A significant difference between the D3 cUL and D30 cUL group is indicated by * in black (** p < 0.01; nested one-way ANOVA and Tukey’s post hoc test).
(EPS)

S5 Fig Chemical unilateral labyrinthectomy does not modulate KCC2 expression in the deafferented lateral vestibular nucleus. A. Confocal immunostaining images of KCC2 neuronal membrane staining (green) in the deafferented lateral vestibular nucleus (LVN) for SHAM group (n = 3), three days (D3 cUL, n = 3) and thirty days (D30 cUL, n = 4) after cUL. Scale bar = 10 µm. B. Quantification of the density of membrane labeling in vestibular neurons of SHAM (grey) and D3 cUL (blue) groups. The histogram represents group means ± SEM, and each point corresponds to the mean number of neuron per animal analyzed.
(EPS)

## Abbreviations

ABR: Auditory brainstem responses
BrdU: bromodeoxyuridine
cUL: chemical unilateral labyrinthectomy
LDBT: light/dark box test
MVN: medial vestibular nuclei
VN: vestibular nuclei
