# Supplementary material for: Central and peripheral plasticity after chemical unilateral labyrinthectomy: Glial responses, calyces dynamics, and behavioral consequences
Source: PLoS One. 2026 Sep 1;21(9):e0355839. doi: 10.1371/journal.pone.0355839 (PMC13533426; doi:10.1371/journal.pone.0355839)
Supplement: Supplementary file 1 [file pone.0355839.s006.docx]

**Reponses to the journal requirements:**

We sincerely thank the Academic Editor for the helpful technical guidance regarding the manuscript formatting. Their comments allowed us to efficiently address all required adjustments. Please find below our responses.

1. Please ensure that your manuscript meets PLOS ONE's style requirements, including those for file naming. The PLOS ONE style templates can be found at

<https://journals.plos.org/plosone/s/file?id=wjVg/PLOSOne_formatting_sample_main_body.pdf> and

<https://journals.plos.org/plosone/s/file?id=ba62/PLOSOne_formatting_sample_title_authors_affiliations.pdf>

Thank you for your comment, we have corrected the affiliations in the manuscript and ensured that the entire manuscript now complies with PLOS ONE formatting guidelines. We have revised the manuscript structure to follow the typical manuscript structure, with the Methodology section placed after the Introduction and before the Results, Discussion, and Conclusion, as recommended.

2. To comply with PLOS One submissions requirements, in your Methods section, please provide additional information regarding the experiments involving animals and ensure you have included details on (1) methods of sacrifice, (2) efforts to alleviate suffering.

We have stated methods on sacrifice (1) in the Methods section, **lines 99-100**: “all animals were perfused at the end of the experiment for immunohistochemical analysis.” And **lines 250-253** “The rats were injected with buprenorphine (Buprecare®, 0.03 mg/kg, s.c.) and deeply anesthetized with a mixture of ketamine 1000 (100mg/kg, i.p) and medetomidine (0,5 mg/kg, i.p) prior to intracardiac perfusion. The intracardiac injection of 400 mL of isotonic saline (0.9% NaCl) was followed by 400 mL of 4% paraformaldehyde in 0.1 M phosphate buffer (PB), pH 7.4.”

We have stated methods on efforts to alleviate suffering (2) in the Methods section, **lines 150-153**: “Post-operatively, animals were monitored daily for 1 week, corresponding to the critical phase of the vestibular syndrome, and high-fat food was provided in the cage to compensate for food intake and support recovery. Body weight and general condition were assessed daily using a standardized clinical scoring grid throughout this period.”

3. Thank you for stating the following financial disclosure: dans la cover letter

“This research was supported by grants from the Ministère de l’Enseignement Supérieur et de la Recherche and CNRS (UMR 7077 Aix Marseille University).”

Please state what role the funders took in the study.  If the funders had no role, please state: "The funders had no role in study design, data collection and analysis, decision to publish, or preparation of the manuscript."

If this statement is not correct you must amend it as needed.

Please include this amended Role of Funder statement in your cover letter; we will change the online submission form on your behalf.

We thank the academic editor for this comment. The following statement has been added to the cover letter as requested “The funders had no role in study design, data collection and analysis, decision to publish, or preparation of the manuscript.”

4. In the online submission form, you indicated that [All data will be made available without any restriction if the manuscript is send to reviewers].

All PLOS journals now require all data underlying the findings described in their manuscript to be freely available to other researchers, either 1. In a public repository, 2. Within the manuscript itself, or 3. Uploaded as supplementary information.

This policy applies to all data except where public deposition would breach compliance with the protocol approved by your research ethics board. If your data cannot be made publicly available for ethical or legal reasons (e.g., public availability would compromise patient privacy), please explain your reasons on resubmission and your exemption request will be escalated for approval.

We thank the reviewer for this clarification. In accordance with the PLOS data availability policy, we have made the raw data underlying the findings reported in this manuscript publicly accessible. The dataset has been uploaded and can be accessed through the following link: <https://figshare.com/s/763b777fdb159b5442b2>.

We have also updated the Data Availability Statement accordingly to reflect the availability of the raw data.

5. We are unable to open your Supporting Information file. Please kindly revise as necessary and re-upload.

We thank the academic editor for bringing this to our attention. We have re-uploaded all Supporting Information files to ensure they are accessible. If the issue persists, please contact us, and we will provide the files through an alternative file-sharing service (e.g., WeTransfer).

If the reviewer comments include a recommendation to cite specific previously published works, please review and evaluate these publications to determine whether they are relevant and should be cited. There is no requirement to cite these works unless the editor has indicated otherwise.

**Reponses to the reviewers**

The authors are grateful to the reviewers for giving us the opportunity to revise and improve the paper while offering their helpful and constructive feedback. We have carefully considered their thoughtful and relevant comments and recommendations. We explain herein how we have revised the paper accordingly. We would like to express our appreciation to the reviewers for taking the time for this revision and for providing us with such insightful guidance. Please find below our responses.

5. Review Comments to the Author

Reviewer #1: The authors present very intersting data on central and peripheral plasticity after chemical unilateral labyrinthecotmy in rats. From my point of view the scientific question and the results are of interest for the readership of PlosOne.

The manuscript is written fluently, clearly structured and conclusive.

I have only minor comments.

To make the present animal study more engaging for the broad readership of *PLOS ONE*—including practicing clinicians—I would suggest to draw a more concrete link to the clinically relevant condition of acute unilateral vestibulopathy in humans. This applies in particular to the Introduction and the Discussion. For instance, how can these results be interpreted in light of the highly variable—and sometimes persistently incomplete—central compensation observed in humans following AUVP?

We thank the reviewer for this valuable suggestion. In response, we have revised both the Introduction and the Discussion to strengthen the translational relevance of our study. The Introduction (**see lines 53-68**) now provides a clearer rationale linking the cUL model to acute unilateral vestibulopathy in humans and highlights the clinical context in which our findings should be interpreted. In addition, we have added a dedicated "Clinical relevance and limitations" section at the end of the Discussion (**lines 779 – 805**). In this section, we discuss how our findings may contribute to understanding the variability of vestibular compensation observed in patients, including the possibility that persistent inflammation and/or altered peripheral plasticity could contribute to incomplete recovery in a subset of individuals. We also explicitly address the limitations of the cUL model with respect to the clinical condition.

Reviewer #2: The abstract lacks specifications in the methodology and results sections. The abstract would benefit from adding the number of subjects and some statistical analysis in the results.

We thank the reviewer for this helpful comment. Following this suggestion, we revised the abstract accordingly (**see lines 17-33**).

In the main manuscript, the methodology is placed after the conclusion, which is confusing.

Thank you for this comment. We now respect the typical manuscript structure with Methodology following Introduction and preceding the Results, Discussion and Conclusion.

The results section would benefit from a paragraph with descriptive statistics.

We thank the reviewer for this suggestion. Descriptive statistics are already provided in the manuscript through the combination of the Methods section and the figures. In accordance with PLOS One guidelines and common reporting practices, the Results section focuses on reporting the statistical values necessary for interpreting the findings, particularly p-values and degrees of freedom for each analysis.

Mean values and SEM are displayed directly in the figures, allowing readers to access the descriptive data alongside the graphical representation of the results. We also report degrees of freedom in the Results section, which we consider sufficient to support interpretation of the inferential analyses.

Given the large number of datasets and comparisons included in the manuscript, adding a separate descriptive statistics table would result in an excessively large and difficult-to-read table, without substantially improving clarity.

No blinding statement for behavioral analysis: For subjective qualitative scoring, this is essential. Not mentioned.
We thank the reviewer for raising this important point. The behavioral analysis in this study focused on well-established and characteristic postural vestibular symptoms following unilateral vestibular lesion, including widening of the support base surface, ipsilesional headtilt, asymmetric muscle tone, and alterations of vestibulo(spinal reflexes such as landing and righting reflexes (Cassel et al., 2018; Chen et al., 2019; Ito et al., 2019; Liberge et al., 2010; Lindner et al., 2019; Rastoldo et al., 2020). These behavioral signs are robust, stereotyped, and widly used for assessing vestibular syndrome severity.

Blinding was not applied during behavioral scoring, as the aim of this study was to compare cUL animals with sham-operated animals. The vestibular deficits induced by our cUL produce a distinct and readily observable behavioral phenotype, allowing clear identification of the experimental groups. Behavioral assessment was performed using predefined criteria based on established literature.

Cassel, R., Bordiga, P., Pericat, D., Hautefort, C., Tighilet, B., Chabbert, C., 2018. New mouse model for inducing and evaluating unilateral vestibular deafferentation syndrome. Journal of Neuroscience Methods 293, 128–135. https://doi.org/10.1016/j.jneumeth.2017.09.002

Chen, Z.-P., Zhang, X.-Y., Peng, S.-Y., Yang, Z.-Q., Wang, Y.-B., Zhang, Y.-X., Chen, X., Wang, J.-J., Zhu, J.-N., 2019. Histamine H1 Receptor Contributes to Vestibular Compensation. J Neurosci 39, 420–433. https://doi.org/10.1523/JNEUROSCI.1350-18.2018

Ito, T., Tatsumi, K., Takimoto, Y., Nishimura, T., Imai, T., Yamanaka, T., Takeda, N., Wanaka, A., Kitahara, T., 2019. Vestibular Compensation after Vestibular Dysfunction Induced by Arsanilic Acid in Mice. Brain Sci 9, 329. https://doi.org/10.3390/brainsci9110329

Liberge, M., Manrique, C., Bernard-Demanze, L., Lacour, M., 2010. Changes in TNFα, NFκB and MnSOD protein in the vestibular nuclei after unilateral vestibular deafferentation. J Neuroinflammation 7, 91. https://doi.org/10.1186/1742-2094-7-91

Lindner, M., Gosewisch, A., Eilles, E., Branner, C., Krämer, A., Oos, R., Wolf, E., Ziegler, S., Bartenstein, P., Brandt, T., Dieterich, M., Zwergal, A., 2019. Ginkgo biloba Extract EGb 761 Improves Vestibular Compensation and Modulates Cerebral Vestibular Networks in the Rat. Front Neurol 10, 147. https://doi.org/10.3389/fneur.2019.00147

Rastoldo, G., Marouane, E., El Mahmoudi, N., Péricat, D., Bourdet, A., Timon-David, E., Dumas, O., Chabbert, C., Tighilet, B., 2020. Quantitative Evaluation of a New Posturo-Locomotor Phenotype in a Rodent Model of Acute Unilateral Vestibulopathy. Frontiers in Neurology 11.

The SHAM group is not a proper control for the behavioral experiments. They have a significant auditory and likely behavioral insult from the surgical procedure itself. A 30 dB hearing loss is not "mild" in the context of sensory processing and can affect posture, locomotion, and anxiety. Any significant difference between the cUL and SHAM groups could be due to the degree of insult rather than to the presence of a specific vestibular lesion. The authors fail to discuss this confound. Their conclusion that cUL specifically causes certain postural or anxiety effects is undermined without a proper SHAM control (e.g., a tympanostomy without perforation or a different surgical approach). This should be stated in a limitations section for this paper.

We thank the reviewer for this important comment and agree that the hearing loss observed in the SHAM group should be considered when interpreting behavioral data. However, the SHAM group was used as a surgical control to account for the effects of anesthesia and middle-ear manipulation, rather than as a model of intact auditory function. Importantly, we did not observe the postural vestibular syndrome in SHAM animals, suggesting that the behavioral deficits observed in cUL animals cannot be explained by tympanic membrane perforation alone.

The SHAM procedure induced an approximately 30 dB auditory threshold shift, which falls within the range classified as slight hearing loss (26–40 dB) (Natarajan et al., 2023; Ryan et al., 2016). Importantly, in SHAM animals, no significant differences were observed at any postoperative time point compared with preoperative values for postural parameters, including support surface, body sway, time spent on the front left paw, and weight distribution on the left paws (Fig. 7). Similarly, no significant changes were observed in locomotor parameters, including rearing time and left circling behavior (Fig. 8), nor in anxiety-related measures, including the number of transitions and time spent in the white compartment (Fig. 9). While several studies in humans have reported an association between hearing loss and postural impairment, these findings mainly concern elderly populations (Nanavati and Jain, 2025; Sakurai et al., 2021), and comparable evidence in rodent models remains limited. Therefore, although a contribution of auditory alteration to some behavioral measures cannot be completely excluded, it is unlikely to account for the marked vestibular and postural phenotype observed after cUL.

We have added this point to the limitations section to acknowledge this potential confounding factor: “In addition, SHAM surgery induced moderate ipsilateral hearing loss due to tympanic membrane perforation. While this may contribute to some behavioral measures, it is unlikely to account for the pronounced vestibular deficits specifically observed after cUL.” (**lines 802-805**).

Ryan, A.F., Kujawa, S.G., Hammill, T., Le Prell, C., Kil, J., 2016. Temporary and Permanent Noise-induced Threshold Shifts: A Review of Basic and Clinical Observations. Otology & Neurotology 37, e271–e275. https://doi.org/10.1097/MAO.0000000000001071

Natarajan, N., Batts, S., Stankovic, K.M., 2023. Noise-Induced Hearing Loss. JCM 12, 2347. https://doi.org/10.3390/jcm12062347

Sakurai, R., Suzuki, H., Ogawa, S., Takahashi, M., Fujiwara, Y., 2021. Hearing loss and increased gait variability among older adults. Gait Posture 87, 54–58. https://doi.org/10.1016/j.gaitpost.2021.04.007

Nanavati, N., Jain, P., 2025. Postural Stability: The Role of Auditory Input in Normal Hearing Individuals and Older Adults with Hearing Loss. Iran J Otorhinolaryngol 37, 151–159. https://doi.org/10.22038/ijorl.2025.81193.3733

The BrdU experiment is underpowered. They used a single BrdU injection at D3 and sacrificed animals 3 hours later to capture acute proliferation (S-phase). Their control for "survival" was a single injection at D3 and sacrifice at D30. A single pulse will only label cells that were in S-phase at that exact 3-hour window. This is highly likely to miss any ongoing, low-level proliferation. To claim "no cell proliferation," they need a more robust method, such as minipump delivery of EdU over several days or multiple BrdU injections across the first week. This should be either explained or stated in the discussion section for this paper.

We thank the reviewer for this insightful comment and agree that a single BrdU pulse labels only cells undergoing S-phase within a restricted time window, which may underestimate low-level or ongoing proliferation. In the present study, this design ensured direct comparability with our previous UVN model (Marouane et al., 2021; Rastoldo et al., 2021, 2020), in which the same BrdU protocol revealed strong proliferation in deafferented vestibular nuclei. This allowed us to determine whether cUL induces a similar proliferative response under identical experimental conditions.

Following the reviewer’s comment, we have revised the manuscript to avoid overinterpretation and replaced “no cell proliferation” with “few cell proliferation” throughout the text. We also clarified in the Discussion that low-level proliferation occurring outside the BrdU labeling window cannot be excluded.

**Lines 434-436** : “Unlike in the unilateral vestibular neurectomy, few cell proliferations, no newly generated survival cells, nor any neuronal KCC2 modulation have been found in this cUL model.”

**Line 653**: “Our cUL model induced few cell proliferation but no survival”

**Lines 658-659**: “Our results contrast with those of (Shaabani et al., 2016) who reported BrdU+ cells after p-arsanilic acid exposure, but their multiple BrdU injections between D11–D15 likely captured a broader window than our single BrdU injection during the critical period, potentially explaining the discrepancy.”

Marouane, E., El Mahmoudi, N., Rastoldo, G., Péricat, D., Watabe, I., Lapôtre, A., Tonetto, A., Xavier, F., Dumas, O., Chabbert, C., Artzner, V., Tighilet, B., 2021. Sensorimotor Rehabilitation Promotes Vestibular Compensation in a Rodent Model of Acute Peripheral Vestibulopathy by Promoting Microgliogenesis in the Deafferented Vestibular Nuclei. Cells 10, 3377. https://doi.org/10.3390/cells10123377

Rastoldo, G., El Mahmoudi, N., Marouane, E., Pericat, D., Watabe, I., Toneto, A., López-Juárez, A., Chabbert, C., Tighilet, B., 2021. Adult and endemic neurogenesis in the vestibular nuclei after unilateral vestibular neurectomy. Progress in Neurobiology 196, 101899. https://doi.org/10.1016/j.pneurobio.2020.101899

Rastoldo, G., Marouane, E., El Mahmoudi, N., Péricat, D., Bourdet, A., Timon-David, E., Dumas, O., Chabbert, C., Tighilet, B., 2020. Quantitative Evaluation of a New Posturo-Locomotor Phenotype in a Rodent Model of Acute Unilateral Vestibulopathy. Frontiers in Neurology 11.

Analysis:

Cells within the same animal are not independent. The better statistical unit is the animal's mean value. Using individual images inflates the sample size artificially and increases the risk of Type I error and causes pseudoreplication.

We thank the reviewer for this comment.

In this study, statistical analyses were performed at the level of the animal, which was the experimental unit. Cell-level measurements were not treated as independent observations; mean values per animal were used for the figures.

Cell counting data were also analyzed using a nested ANOVA with cells nested within animals, as stated in the Method section: “The statistical analyses of cellular data in the deafferented MVN were evaluated by nested one-way ANOVA” (**lines 352-353**).

Grossly underpowered SHAM ABR – n=2 for SHAM. Cannot make meaningful comparisons.

We thank the reviewer for this comment. We agree that the SHAM group size (n = 2) is not sufficient for statistical comparisons. Accordingly, no statistical analyses were performed between cUL and SHAM groups, nor within the SHAM group. Statistical comparisons were restricted to within the cUL group, comparing preoperative, D3, and D30 time points for left and right ears (n = 4 animals), as stated in the manuscript.

KCC2 only at D3 – No D30 data. Changes could occur later; absence at D3 does prove no effect.

We thank the reviewer for this comment and fully agree that changes in KCC2 expression could occur at later time points and would not be captured at D3 alone. In response, we have added and quantified KCC2 expression at D30 in the cUL condition. As for the D3 cUL group, no difference was observed compared to the sham-operated group (see Fig. S4).

Section 2.2. Unquantified histology – "Disorganization" is a subjective visual description. No cell counts, no scoring system, no statistics.

We thank the reviewer for this comment. We agree that the term “disorganization” is descriptive and not based on a quantitative scoring system. We have revised the wording accordingly to make this clearer in the manuscript. The histological observations are reported to document that the cUL procedure induces structural alterations in the ipsilateral cochlea, in contrast to the contralateral side and SHAM animals. Although the model is primarily used as a unilateral vestibular deafferentation model, we considered it important to report these cochlear changes, as they are sometimes not explicitly described in the literature for “vestibular-only” lesion models. We have clarified the description in the revised text to avoid subjective terminology while preserving this observation, **see lines 398-400** “These alterations affect hair cell afferents (NKAα3+) and nuclei (DAPI) compared with the SHAM group. Although no cell quantification or formal scoring was performed for this descriptive observation, the ipsilateral cochlea consistently appears altered relative to SHAM group.”
