# Supplementary material for: Central and peripheral plasticity after chemical unilateral labyrinthectomy: Glial responses, calyces dynamics, and behavioral consequences
Source: PLoS One. 2026 Sep 1;21(9):e0355839. doi: 10.1371/journal.pone.0355839 (PMC13533426; doi:10.1371/journal.pone.0355839)
Supplement: Peer review history

# Peer review: Decision Letter 0

**DOI:** 10.1371/journal.pone.0355839.r001

**Authors:** Gauri Mankekar

27 May 2026

Dear Dr. Trico,

Thank you for submitting your manuscript to PLOS ONE. After careful consideration, we feel that it has merit but does not fully meet PLOS ONE’s publication criteria as it currently stands. Therefore, we invite you to submit a revised version of the manuscript that addresses the points raised during the review process.

==============================

**ACADEMIC EDITOR: I agree with the reviewers. The typical manuscript is structured with Methodology following Introduction and preceeding the Results, Discussion and Conclusion.** **Please see reviewer's comments, revise and resubmit the manuscript per Plos One guidelines.**

==============================

Please submit your revised manuscript by Jul 11 2026 11:59PM. If you will need more time than this to complete your revisions, please reply to this message or contact the journal office at plosone@plos.org. When you're ready to submit your revision, log on to https://www.editorialmanager.com/pone/ and select the 'Submissions Needing Revision' folder to locate your manuscript file.

- A letter that responds to each point raised by the academic editor and reviewer(s). You should upload this letter as a separate file labeled 'Response to Reviewers'.
- A marked-up copy of your manuscript that highlights changes made to the original version. You should upload this as a separate file labeled 'Revised Manuscript with Track Changes'.
- An unmarked version of your revised paper without tracked changes. You should upload this as a separate file labeled 'Manuscript'.

If you would like to make changes to your financial disclosure, please include your updated statement in your cover letter. Guidelines for resubmitting your figure files are available below the reviewer comments at the end of this letter.

If applicable, we recommend that you deposit your laboratory protocols in protocols.io to enhance the reproducibility of your results. Protocols.io assigns your protocol its own identifier (DOI) so that it can be cited independently in the future. For instructions see: https://journals.plos.org/plosone/s/submission-guidelines#loc-laboratory-protocols. Additionally, PLOS ONE offers an option for publishing peer-reviewed Lab Protocol articles, which describe protocols hosted on protocols.io. Read more information on sharing protocols at https://plos.org/protocols?utm_medium=editorial-email&utm_source=authorletters&utm_campaign=protocols.

As the corresponding author, your ORCID iD is verified in the submission system and will appear in the published article. PLOS supports the use of ORCID, and we encourage all coauthors to register for an ORCID iD and use it as well. Please encourage your coauthors to verify their ORCID iD within the submission system before final acceptance, as unverified ORCID iDs will not appear in the published article. *Only*  the individual author can complete the verification step; PLOS staff *cannot*  verify ORCID iDs on behalf of authors.

We look forward to receiving your revised manuscript.

Kind regards,

Gauri Mankekar, MD,PhD,FACS

Academic Editor

PLOS One

Journal requirements:

When submitting your revision, we need you to address these additional requirements.

1. Please ensure that your manuscript meets PLOS ONE's style requirements, including those for file naming. The PLOS ONE style templates can be found at

https://journals.plos.org/plosone/s/file?id=wjVg/PLOSOne_formatting_sample_main_body.pdf and

https://journals.plos.org/plosone/s/file?id=ba62/PLOSOne_formatting_sample_title_authors_affiliations.pdf

2. To comply with PLOS One submissions requirements, in your Methods section, please provide additional information regarding the experiments involving animals and ensure you have included details on (1) methods of sacrifice, (2) efforts to alleviate suffering.

3. Thank you for stating the following financial disclosure:

“This research was supported by grants from the Ministère de l’Enseignement Supérieur et de la Recherche and CNRS (UMR 7077 Aix Marseille University).”

Please state what role the funders took in the study.  If the funders had no role, please state: "The funders had no role in study design, data collection and analysis, decision to publish, or preparation of the manuscript."

If this statement is not correct you must amend it as needed.

Please include this amended Role of Funder statement in your cover letter; we will change the online submission form on your behalf.

4. In the online submission form, you indicated that [All data will be made available without any restriction if the manuscript is send to reviewers].

All PLOS journals now require all data underlying the findings described in their manuscript to be freely available to other researchers, either 1. In a public repository, 2. Within the manuscript itself, or 3. Uploaded as supplementary information.

This policy applies to all data except where public deposition would breach compliance with the protocol approved by your research ethics board. If your data cannot be made publicly available for ethical or legal reasons (e.g., public availability would compromise patient privacy), please explain your reasons on resubmission and your exemption request will be escalated for approval.

5. We are unable to open your Supporting Information file. Please kindly revise as necessary and re-upload.

If the reviewer comments include a recommendation to cite specific previously published works, please review and evaluate these publications to determine whether they are relevant and should be cited. There is no requirement to cite these works unless the editor has indicated otherwise.

[Note: HTML markup is below. Please do not edit.]

Reviewers' comments:

Reviewer's Responses to Questions

**Comments to the Author**

1. Is the manuscript technically sound, and do the data support the conclusions?

Reviewer #1: Yes

Reviewer #2: Partly

**********

2. Has the statistical analysis been performed appropriately and rigorously?

Reviewer #1: Yes

Reviewer #2: I Don't Know

**********

3. Have the authors made all data underlying the findings in their manuscript fully available?

The PLOS Data policy

Reviewer #1: Yes

Reviewer #2: Yes

**********

4. Is the manuscript presented in an intelligible fashion and written in standard English?

Reviewer #1: Yes

Reviewer #2: Yes

**********

Reviewer #1: The authors present very intersting data on central and peripheral plasticity after chemical unilateral labyrinthecotmy in rats. From my point of view the scientific question and the results are of interest for the readership of PlosOne.

The manuscript is written fluently, clearly structured and conclusive.

I have only minor comments.

To make the present animal study more engaging for the broad readership of *PLOS ONE*—including practicing clinicians—I would suggest to draw a more concrete link to the clinically relevant condition of acute unilateral vestibulopathy in humans. This applies in particular to the Introduction and the Discussion. For instance, how can these results be interpreted in light of the highly variable—and sometimes persistently incomplete—central compensation observed in humans following AUVP?

Reviewer #2: The abstract lacks specifications in the methodology and results sections. The abstract would benefit from adding the number of subjects and some statistical analysis in the results.

In the main manuscript, the methodology is placed after the conclusion, which is confusing.

The results section would benefit from a paragraph with descriptive statistics.

No blinding statement for behavioral analysis: For subjective qualitative scoring, this is essential. Not mentioned.

The SHAM group is not a proper control for the behavioral experiments. They have a significant auditory and likely behavioral insult from the surgical procedure itself. A 30 dB hearing loss is not "mild" in the context of sensory processing and can affect posture, locomotion, and anxiety. Any significant difference between the cUL and SHAM groups could be due to the degree of insult rather than to the presence of a specific vestibular lesion. The authors fail to discuss this confound. Their conclusion that cUL specifically causes certain postural or anxiety effects is undermined without a proper SHAM control (e.g., a tympanostomy without perforation or a different surgical approach).

This should be stated in a limitations section for this paper.

The BrdU experiment is underpowered. They used a single BrdU injection at D3 and sacrificed animals 3 hours later to capture acute proliferation (S-phase). Their control for "survival" was a single injection at D3 and sacrifice at D30. A single pulse will only label cells that were in S-phase at that exact 3-hour window. This is highly likely to miss any ongoing, low-level proliferation. To claim "no cell proliferation," they need a more robust method, such as minipump delivery of EdU over several days or multiple BrdU injections across the first week. This should be either explained or stated in the discussion section for this paper.

Analysis:

Cells within the same animal are not independent. The better statistical unit is the animal's mean value. Using individual images inflates the sample size artificially and increases the risk of Type I error and causes pseudoreplication.

Grossly underpowered SHAM ABR – n=2 for SHAM. Cannot make meaningful comparisons.

KCC2 only at D3 – No D30 data. Changes could occur later; absence at D3 does prove no effect.

Section 2.2. Unquantified histology – "Disorganization" is a subjective visual description. No cell counts, no scoring system, no statistics.

**********

what does this mean?). If published, this will include your full peer review and any attached files.

If you choose “no”, your identity will remain anonymous but your review may still be made public.

**Do you want your identity to be public for this peer review?**  For information about this choice, including consent withdrawal, please see our Privacy Policy

Reviewer #1: No

Reviewer #2: **Yes:**  Isra Aljazeeri

**********

[NOTE: If reviewer comments were submitted as an attachment file, they will be attached to this email and accessible via the submission site. Please log into your account, locate the manuscript record, and check for the action link "View Attachments". If this link does not appear, there are no attachment files.]

To ensure your figures meet our technical requirements, please review our figure guidelines: https://journals.plos.org/plosone/s/figures

You may also use PLOS’s free figure tool, NAAS, to help you prepare publication quality figures: https://journals.plos.org/plosone/s/figures#loc-tools-for-figure-preparation.

NAAS will assess whether your figures meet our technical requirements by comparing each figure against our figure specifications.

# Author comment: Author response to Decision Letter 1

**DOI:** 10.1371/journal.pone.0355839.r002

14 Jul 2026

Reponses to the journal requirements:

We sincerely thank the Academic Editor for the helpful technical guidance regarding the manuscript formatting. Their comments allowed us to efficiently address all required adjustments. Please find below our responses.

1. Please ensure that your manuscript meets PLOS ONE's style requirements, including those for file naming. The PLOS ONE style templates can be found at

https://journals.plos.org/plosone/s/file?id=wjVg/PLOSOne_formatting_sample_main_body.pdf and

https://journals.plos.org/plosone/s/file?id=ba62/PLOSOne_formatting_sample_title_authors_affiliations.pdf

Thank you for your comment, we have corrected the affiliations in the manuscript and ensured that the entire manuscript now complies with PLOS ONE formatting guidelines. We have revised the manuscript structure to follow the typical manuscript structure, with the Methodology section placed after the Introduction and before the Results, Discussion, and Conclusion, as recommended.

2. To comply with PLOS One submissions requirements, in your Methods section, please provide additional information regarding the experiments involving animals and ensure you have included details on (1) methods of sacrifice, (2) efforts to alleviate suffering.

We have stated methods on sacrifice (1) in the Methods section, lines 99-100: “all animals were perfused at the end of the experiment for immunohistochemical analysis.” And lines 250-253 “The rats were injected with buprenorphine (Buprecare®, 0.03 mg/kg, s.c.) and deeply anesthetized with a mixture of ketamine 1000 (100mg/kg, i.p) and medetomidine (0,5 mg/kg, i.p) prior to intracardiac perfusion. The intracardiac injection of 400 mL of isotonic saline (0.9% NaCl) was followed by 400 mL of 4% paraformaldehyde in 0.1 M phosphate buffer (PB), pH 7.4.”

We have stated methods on efforts to alleviate suffering (2) in the Methods section, lines 150-153: “Post-operatively, animals were monitored daily for 1 week, corresponding to the critical phase of the vestibular syndrome, and high-fat food was provided in the cage to compensate for food intake and support recovery. Body weight and general condition were assessed daily using a standardized clinical scoring grid throughout this period.”

3. Thank you for stating the following financial disclosure: dans la cover letter

“This research was supported by grants from the Ministère de l’Enseignement Supérieur et de la Recherche and CNRS (UMR 7077 Aix Marseille University).”

Please state what role the funders took in the study. If the funders had no role, please state: "The funders had no role in study design, data collection and analysis, decision to publish, or preparation of the manuscript."

If this statement is not correct you must amend it as needed.

Please include this amended Role of Funder statement in your cover letter; we will change the online submission form on your behalf.

We thank the academic editor for this comment. The following statement has been added to the cover letter as requested “The funders had no role in study design, data collection and analysis, decision to publish, or preparation of the manuscript.”

4. In the online submission form, you indicated that [All data will be made available without any restriction if the manuscript is send to reviewers].

All PLOS journals now require all data underlying the findings described in their manuscript to be freely available to other researchers, either 1. In a public repository, 2. Within the manuscript itself, or 3. Uploaded as supplementary information.

This policy applies to all data except where public deposition would breach compliance with the protocol approved by your research ethics board. If your data cannot be made publicly available for ethical or legal reasons (e.g., public availability would compromise patient privacy), please explain your reasons on resubmission and your exemption request will be escalated for approval.

We thank the reviewer for this clarification. In accordance with the PLOS data availability policy, we have made the raw data underlying the findings reported in this manuscript publicly accessible. The dataset has been uploaded and can be accessed through the following link: https://figshare.com/s/763b777fdb159b5442b2.

We have also updated the Data Availability Statement accordingly to reflect the availability of the raw data.

5. We are unable to open your Supporting Information file. Please kindly revise as necessary and re-upload.

We thank the academic editor for bringing this to our attention. We have re-uploaded all Supporting Information files to ensure they are accessible. If the issue persists, please contact us, and we will provide the files through an alternative file-sharing service (e.g., WeTransfer).

If the reviewer comments include a recommendation to cite specific previously published works, please review and evaluate these publications to determine whether they are relevant and should be cited. There is no requirement to cite these works unless the editor has indicated otherwise.

Reponses to the reviewers

The authors are grateful to the reviewers for giving us the opportunity to revise and improve the paper while offering their helpful and constructive feedback. We have carefully considered their thoughtful and relevant comments and recommendations. We explain herein how we have revised the paper accordingly. We would like to express our appreciation to the reviewers for taking the time for this revision and for providing us with such insightful guidance. Please find below our responses.

5. Review Comments to the Author

Reviewer #1: The authors present very intersting data on central and peripheral plasticity after chemical unilateral labyrinthecotmy in rats. From my point of view the scientific question and the results are of interest for the readership of PlosOne.

The manuscript is written fluently, clearly structured and conclusive.

I have only minor comments.

To make the present animal study more engaging for the broad readership of *PLOS ONE*—including practicing clinicians—I would suggest to draw a more concrete link to the clinically relevant condition of acute unilateral vestibulopathy in humans. This applies in particular to the Introduction and the Discussion. For instance, how can these results be interpreted in light of the highly variable—and sometimes persistently incomplete—central compensation observed in humans following AUVP?

We thank the reviewer for this valuable suggestion. In response, we have revised both the Introduction and the Discussion to strengthen the translational relevance of our study. The Introduction (see lines 53-68) now provides a clearer rationale linking the cUL model to acute unilateral vestibulopathy in humans and highlights the clinical context in which our findings should be interpreted. In addition, we have added a dedicated "Clinical relevance and limitations" section at the end of the Discussion (lines 779 – 805). In this section, we discuss how our findings may contribute to understanding the variability of vestibular compensation observed in patients, including the possibility that persistent inflammation and/or altered peripheral plasticity could contribute to incomplete recovery in a subset of individuals. We also explicitly address the limitations of the cUL model with respect to the clinical condition.

Reviewer #2: The abstract lacks specifications in the methodology and results sections. The abstract would benefit from adding the number of subjects and some statistical analysis in the results.

We thank the reviewer for this helpful comment. Following this suggestion, we revised the abstract accordingly (see lines 17-33).

In the main manuscript, the methodology is placed after the conclusion, which is confusing.

Thank you for this comment. We now respect the typical manuscript structure with Methodology following Introduction and preceding the Results, Discussion and Conclusion.

The results section would benefit from a paragraph with descriptive statistics.

We thank the reviewer for this suggestion. Descriptive statistics are already provided in the manuscript through the combination of the Methods section and the figures. In accordance with PLOS One guidelines and common reporting practices, the Results section focuses on reporting the statistical values necessary for interpreting the findings, particularly p-values and degrees of freedom for each analysis.

Mean values and SEM are displayed directly in the figures, allowing readers to access the descriptive data alongside the graphical representation of the results. We also report degrees of freedom in the Results section, which we consider sufficient to support interpretation of the inferential analyses.

Given the large number of datasets and comparisons included in the manuscript, adding a separate descriptive statistics table would result in an excessively large and difficult-to-read table, without substantially improving clarity.

No blinding statement for behavioral analysis: For subjective qualitative scoring, this is essential. Not mentioned.

We thank the reviewer for raising this important point. The behavioral analysis in this study focused on well-established and characteristic postural vestibular symptoms following unilateral vestibular lesion, including widening of the support base surface, ipsilesional headtilt, asymmetric muscle tone, and alterations of vestibulo(spinal reflexes such as landing and righting reflexes (Cassel et al., 2018; Chen et al., 2019; Ito et al., 2019; Liberge et al., 2010; Lindner et al., 2019; Rastoldo et al., 2020). These behavioral signs are robust, stereotyped, and widly used for assessing vestibular syndrome severity.

Blinding was not applied during behavioral scoring, as the aim of this study was to compare cUL animals with sham-operated animals. The vestibular deficits induced by our cUL produce a distinct and readily observable behavioral phenotype, allowing clear identification of the experimental groups. Behavioral assessment was performed using predefined criteria based on established literature.

Cassel, R., Bordiga, P., Pericat, D., Hautefort, C., Tighilet, B., Chabbert, C., 2018. New mouse model for inducing and evaluating unilateral vestibular deafferentation syndrome. Journal of Neuroscience Methods 293, 128–135. https://doi.org/10.1016/j.jneumeth.2017.09.002

Chen, Z.-P., Zhang, X.-Y., Peng, S.-Y., Yang, Z.-Q., Wang, Y.-B., Zhang, Y.-X., Chen, X., Wang, J.-J., Zhu, J.-N., 2019. Histamine H1 Receptor Contributes to Vestibular Compensation. J Neurosci 39, 420–433. https://doi.org/10.1523/JNEUROSCI.1350-18.2018

Ito, T., Tatsumi, K., Takimoto, Y., Nishimura, T., Imai, T., Yamanaka, T., Takeda, N., Wanaka, A., Kitahara, T., 2019. Vestibular Compensation after Vestibular Dysfunction Induced by Arsanilic Acid in Mice. Brain Sci 9, 329. https://doi.org/10.3390/brainsci9110329

Liberge, M., Manrique, C., Bernard-Demanze, L., Lacour, M., 2010. Changes in TNFα, NFκB and MnSOD protein in the vestibular nuclei after unilateral vestibular deafferentation. J Neuroinflammation 7, 91. https://doi.org/10.1186/1742-2094-7-91

Lindner, M., Gosewisch, A., Eilles, E., Branner, C., Krämer, A., Oos, R., Wolf, E., Ziegler, S., Bartenstein, P., Brandt, T., Dieterich, M., Zwergal, A., 2019. Ginkgo biloba Extract EGb 761 Improves Vestibular Compensation and Modulates Cerebral Vestibular Networks in the Rat. Front Neurol 10, 147. https://doi.org/10.3389/fneur.2019.00147

Rastoldo, G., Marouane, E., El Mahmoudi, N., Péricat, D., Bourdet, A., Timon-David, E., Dumas, O., Chabbert, C., Tighilet, B., 2020. Quantitative Evaluation of a New Posturo-Locomotor Phenotype in a Rodent Model of Acute Unilateral Vestibulopathy. Frontiers in Neurology 11.

The SHAM group is not a proper control for the behavioral experiments. They have a significant auditory and likely behavioral insult from the surgical procedure itself. A 30 dB hearing loss is not "mild" in the context of sensory processing and can affect posture, locomotion, and anxiety. Any significant difference between the cUL and SHAM groups could be due to the degree of insult rather than to the presence of a specific vestibular lesion. The authors fail to discuss this confound. Their conclusion that cUL specifically causes certain postural or anxiety effects is undermined without a proper SHAM control (e.g., a tympanostomy without perforation or a different surgical approach). This should be stated in a limitations section for this paper.

We thank the reviewer for this important comment and agree that the hearing loss observed in the SHAM group should be considered when interpreting behavioral data. However, the SHAM group was used as a surgical control to account for the effects of anesthesia and middle-ear manipulation, rather than as a model of intact auditory function. Importantly, we did not observe the postural vestibular syndrome in SHAM animals, suggesting that the behavioral deficits observed in cUL animals cannot be explained by tympanic membrane perforation alone.

The SHAM procedure induced an approximately 30 dB auditory threshold shift, which falls within the range classified as slight hearing loss (26–40 dB) (Natarajan et al., 2023; Ryan et al., 2016). Importantly, in SHAM animals, no significant differences were observed at any postoperative time point compared with preoperative values for postural parameters, including support surface, body sway, time spent on the front left paw, and weight distribution on the left paws (Fig. 7). Similarly, no significant changes were observed in locomotor parameters, including rearing time and left circling behavior (Fig. 8), nor in anxiety-related measures, including the number of transitions and time spent in the white compartment (Fig. 9). While several studies in humans have reported an association between hearing loss and postural impairment, these findings mainly concern elderly populations (Nanavati and Jain, 2025; Sakurai et al., 2021), and comparable evidence in rodent models remains limited. Therefore, although a contribution of auditory alteration to some behavioral measures cannot be completely excluded, it is unlikely to account for the marked vestibular and postural phenotype observed after cUL.

We have added this point to the limitations section to acknowledge this potential confounding factor: “In addition, SHAM surgery induced moderate ipsilateral hearing loss due to tympanic membrane perforation. While this may contribute to some behavioral measures, it is unlikely to account for the pronounced vestibular deficits specifically observed after cUL.” (lines 802-805).

Ryan, A.F., Kujawa, S.G., Hammill, T., Le Prell, C., Kil, J., 2016. Temporary and Permanent Noise-induced Threshold Shifts: A Review of Basic and Clinical Observations. Otology & Neurotology 37, e271–e275. https://doi.org/10.1097/MAO.0000000000001071

Natarajan, N., Batts, S., Stankovic, K.M., 2023. Noise-Induced Hearing Loss. JCM 12, 2347. https://doi.org/10.3390/jcm12062347

Sakurai, R., Suzuki, H., Ogawa, S., Takahashi, M., Fujiwara, Y., 2021. Hearing loss and increased gait variability among older adults. Gait Posture 87, 54–58. https://doi.org/10.1016/j.gaitpost.2021.04.007

Nanavati, N., Jain, P., 2025. Postural Stability: The Role of Auditory Input in Normal Hearing Individuals and Older Adults with Hearing Loss. Iran J Otorhinolaryngol 37, 151–159. https://doi.org/10.22038/ijorl.2025.81193.3733

The BrdU experiment is underpowered. They used a single BrdU injection at D3 and sacrificed animals 3 hours later to capture acute proliferation (S-phase). Their control for "survival" was a single injection at D3 and sacrifice at D30. A single pulse will only label cells that were in S-phase at that exact 3-hour window. This is highly likely to miss any ongoing, low-level proliferation. To claim "no cell proliferation," they need a more robust method, such as minipump delivery of EdU over several days or multiple BrdU injections across the first week. This should be either explained or stated in the discussion section for this paper.

We thank the reviewer for this insightful comment and agree that a single BrdU pulse labels only cells undergoing S-phase within a restricted time window, which may

Attachment Submitted filename: Response to Reviewers.docx

# Peer review: Decision Letter 1

**DOI:** 10.1371/journal.pone.0355839.r003

**Authors:** Gauri Mankekar

28 Jul 2026

Central and peripheral plasticity after chemical unilateral labyrinthectomy: glial responses, calyces dynamics, and behavioral consequences

PONE-D-26-17747R1

Dear Dr. Jessica Trico,

We’re pleased to inform you that your manuscript has been judged scientifically suitable for publication and will be formally accepted for publication once it meets all outstanding technical requirements.

Within one week, you’ll receive an e-mail detailing the required amendments. When these have been addressed, you’ll receive a formal acceptance letter and your manuscript will be scheduled for publication.

An invoice will be generated when your article is formally accepted. Please note, if your institution has a publishing partnership with PLOS and your article meets the relevant criteria, all or part of your publication costs will be covered. Please make sure your user information is up-to-date by logging into Editorial Manager at Editorial Manager® and clicking the ‘Update My Information' link at the top of the page. For questions related to billing, please contact billing support.

If your institution or institutions have a press office, please notify them about your upcoming paper to help maximize its impact. If they’ll be preparing press materials, please inform our press team as soon as possible -- no later than 48 hours after receiving the formal acceptance. Your manuscript will remain under strict press embargo until 2 pm Eastern Time on the date of publication. For more information, please contact onepress@plos.org.

Kind regards,

Gauri Mankekar, MD,PhD,FACS

Academic Editor

PLOS One

Additional Editor Comments (optional):

Reviewers' comments:

Reviewer's Responses to Questions

**Comments to the Author**

Reviewer #1: All comments have been addressed

**********

2. Is the manuscript technically sound, and do the data support the conclusions?

Reviewer #1: Yes

**********

3. Has the statistical analysis been performed appropriately and rigorously?

Reviewer #1: Yes

**********

4. Have the authors made all data underlying the findings in their manuscript fully available?

The PLOS Data policy

Reviewer #1: Yes

**********

5. Is the manuscript presented in an intelligible fashion and written in standard English?

Reviewer #1: Yes

**********

Reviewer #1: (No Response)

**********

what does this mean?). If published, this will include your full peer review and any attached files.

If you choose “no”, your identity will remain anonymous but your review may still be made public.

**Do you want your identity to be public for this peer review?**  For information about this choice, including consent withdrawal, please see our Privacy Policy

Reviewer #1: No

**********

# Editor report: Acceptance letter

**DOI:** 10.1371/journal.pone.0355839.r004

**Authors:** Gauri Mankekar

PONE-D-26-17747R1

PLOS One

Dear Dr. Trico,

I'm pleased to inform you that your manuscript has been deemed suitable for publication in PLOS One. Congratulations! Your manuscript is now being handed over to our production team.

At this stage, our production department will prepare your paper for publication. This includes ensuring the following:

* All references, tables, and figures are properly cited

* All relevant supporting information is included in the manuscript submission,

* There are no issues that prevent the paper from being properly typeset

You will receive further instructions from the production team, including instructions on how to review your proof when it is ready. Please keep in mind that we are working through a large volume of accepted articles, so please give us a few days to review your paper and let you know the next and final steps.

Lastly, if your institution or institutions have a press office, please let them know about your upcoming paper now to help maximize its impact. If they'll be preparing press materials, please inform our press team within the next 48 hours. Your manuscript will remain under strict press embargo until 2 pm Eastern Time on the date of publication. For more information, please contact onepress@plos.org.

You will receive an invoice from PLOS for your publication fee after your manuscript has reached the completed accept phase. If you receive an email requesting payment before acceptance or for any other service, this may be a phishing scheme. Learn how to identify phishing emails and protect your accounts at https://explore.plos.org/phishing.

If we can help with anything else, please email us at customercare@plos.org.

Thank you for submitting your work to PLOS One and supporting open access.

Kind regards,

PLOS ONE Editorial Office Staff

on behalf of

Dr. Gauri Mankekar

Academic Editor

PLOS One
